# Organic Fluorescent Sensors for Environmental Analysis: A Critical Review and Insights into Inorganic Alternatives

**DOI:** 10.3390/nano15191512

**Published:** 2025-10-02

**Authors:** Katia Buonasera, Maurilio Galletta, Massimo Rosario Calvo, Gianni Pezzotti Escobar, Antonio Alessio Leonardi, Alessia Irrera

**Affiliations:** 1 CNR IMM-ME, Institute for Microelectronics and Microsystems, Viale F.S. d’Alcontres 31, I-98166 Messina, Italy; katia.buonasera@cnr.it (K.B.); maurilio.galletta@cnr.it (M.G.); alessia.irrera@cnr.it (A.I.); 2 Department of Chemical, Biological, Pharmaceutical, and Environmental Sciences (ChiBioFarAm), University of Messina, Viale F.S. d’Alcontres 31, I-98166 Messina, Italy; massimo.calvo@unime.it; 3 Department of Mathematical and Computer Sciences, Physical Sciences, and Earth Sciences (MIFT), University of Messina, Viale F.S. d’Alcontres 31, I-98166 Messina, Italy

**Keywords:** fluorescent sensors, organic sensors, biosensors, inorganic sensors

## Abstract

The exponential increase in environmental pollutants due to industrialization, urbanization, and agricultural intensification has underscored the urgent need for sensitive, selective, and real-time monitoring technologies. Among emerging analytical tools, organic fluorescent sensors have demonstrated exceptional potential for detecting a wide range of pollutants in water, air, and soil, with a limit of detection (LOD) in the pM–µM range. This review critically examines recent advances in organic fluorescent sensors, focusing on their photophysical properties, molecular structures, sensing mechanisms, and environmental applications. Key categories of organic sensors, including small molecules, polymeric materials, and nanoparticle-based systems, are discussed, highlighting their advantages, such as biocompatibility, tunability, and cost-effectiveness. Comparative insights into inorganic fluorescent sensors, including quantum dots, are also provided, emphasizing their superior photostability and wide operating range (in some cases from pg/mL up to mg/mL) but limited biodegradability and higher toxicity. The integration of nanomaterials and microfluidic systems is presented as a promising route for developing portable, on-site sensing platforms. Finally, the review outlines current challenges and future perspectives, suggesting that fluorescent sensors, particularly organic ones, represent a crucial strategy toward sustainable environmental monitoring and pollutant management.

## 1. Introduction

During the past few years, the continuous growth of industry, urbanization, agricultural intensification, and an energy production system mostly revolving on non-renewable energy sources (oil, coal, natural gas, etc.) has resulted in an exponential increase in environmental pollutants [1,2,3]. Some of the most worrying are volatile organic compounds (VOCs) [4], heavy metals [5], persistent organic pollutants (POPs) [6], and microplastics [7]. The monitoring of these widespread contaminants, as well as their remotion, has become a serious challenge, threatening ecosystems and public health [8,9,10]. These pollutants commonly accumulate in water, soil, and air [11,12,13,14], demanding advanced sensing technologies for effective monitoring and mitigation strategies.

Water pollution is a significant global concern due to the scarcity of drinkable water and the impact that this has on different countries. In addition to posing serious environmental and public health risks, poor water quality incurs substantial economic losses. According to the World Bank Annual Report 2024 [15], when the biological oxygen demand (an analytical parameter representing the degree of organic water pollution) crosses a threshold of 8 milligrams per liter, GDP growth in downstream regions drops by 0.83 percentage points, which equates to around a third of the mean growth rate of 2.33%. Another important point described in the report is the effect of nitrogen coming from agricultural fertilizers and eventually entering rivers, lakes, and oceans. Early exposure of children to nitrates affects their growth and brain development, reducing their health and earning potential as adults. It seems that, for every additional kilogram of nitrogen fertilizer per hectare, childhood stunting can increase by up to 19%, and future adult earnings can fall by up to 2%. Water pollution also negatively impacts the tourism industry, with losses of close to USD 1 billion each year due to losses in fishing and boating activities as a result of nutrient pollution and harmful algal blooms. Commercial fishing and shellfish industries are also hurt by these blooms, which kill fish and contaminate shellfish, resulting in tens of millions of dollars in annual losses. Clean water can increase nearby property values by up to 25%, while polluted water depresses real estate markets. The financial burden associated with poor water quality underscores the fact that safeguarding water resources has become not only an environmental imperative but also a critical strategy for sustainable economic development. Mitigation strategies, such as upgrading wastewater infrastructure and enforcing pollution controls, are therefore economically beneficial: according to the European Central Bank, ecosystem services related to clean water alone generated EUR 234 billion annually across the EU-28 in 2019 [16].

Water pollutants include a wide array of contaminants, such as pathogenic microorganisms, toxic organic compounds, and heavy metals [17]. Some of the most diffused and concerning organic pollutants that contaminate aquatic ecosystems are polycyclic aromatic hydrocarbons (PAHs) and pharmaceutical residues due to industrial and agricultural waste [18]. Moreover, real-time monitoring is also required for harmful algal blooms, which are known to release toxins (e.g., microcystins and saxitoxins) that are detrimental to both human health and marine ecosystems.

Air pollution, contributing to public health issues [19], is generally characterized by VOCs, nitrogen oxides (NO_x_)—typical of high-temperature combustion processes—and particulate matter. Moreover, in this field, real-time detection enabling the constant surveillance of atmospheric pollutants for air quality assessments is critically demanded.

Soil pollution is commonly related to waste treatment, such as industrial refuse, heavy metal accumulation, pesticides in agriculture, and so on [20]. This represents another critical challenge for the environment, where the precise detection, quantification, and mapping of contaminated sites is crucial for land use planning, mitigation strategies, and targeted interventions.

Conventional detection methods for environmental pollutants include gas chromatography coupled to mass spectrometry (GC-MS), high-performance liquid chromatography (HPLC), and spectrophotometry analysis, which guarantee a highly accurate analysis. However, these analysis methods typically require sample preparation, expensive equipment, and specialized laboratories with expert personnel, making them less viable for real-time and spread monitoring. The demand for sensing platforms equipped with portable capabilities, enabling both instant results and on-site field monitoring, is therefore rising. Many of these platforms are designed to be disposable, minimizing costs and enhancing accessibility for broader applications. The ability to swiftly and cost-effectively detect the ever-growing number of environmentally relevant analytes makes them indispensable for pollution control, while their exceptional sensitivity and low detection limits ensure the measurement of trace contaminants while reinforcing safety and regulatory compliance [21].

Among the various sensor technologies developed, fluorescent sensors stand out as some of the most extensively studied. They offer greater robustness than electrical sensors and are immune to electrical noise [22], and their ability to deliver rapid, highly sensitive, and selective detection [23] makes them particularly suitable for real-time environmental monitoring [24]. Fluorescence, observed in numerous natural and synthetic molecules, occurs when electrons absorb low-wavelength light, become excited, and then return to their ground states by emitting light of a longer wavelength [25]. Fluorescence-based sensors can detect changes in intensity, absorption/emission wavelengths, Förster resonance energy transfer (FRET), fluorescence anisotropy, lifetime, and decay time [26]. Among the possible photophysical mechanisms for optical sensors, FRET is one of the most investigated. This mechanism relies on a distance-dependent physical process in which energy is transferred non-radiatively from an excited donor fluorophore to a nearby acceptor fluorescence species. This phenomenon typically occurs over a range of 1–10 nm, revealing molecular interactions and conformational changes at the nanoscale. FRET efficiency is influenced by the spectral overlap, the quantum yield of the donor, and the relative orientation and distance between the donor and acceptor. There are several energy transfer mechanisms other than FRET; however, a detailed discussion of these is beyond the scope of this review.

Their high selectivity, essential for environmental applications, is achieved by conjugating fluorophores with specific recognition elements such as aptamers, chelating ligands, molecularly imprinted polymers (MIPs), antibodies, and enzymes [27]. These functionalization strategies have proven effective in detecting a wide range of pollutants, including heavy metal ions like lead (Pb^2+^) and mercury (Hg^2+^) [28], as well as organic contaminants such as industrial dyes, endocrine-disrupting compounds (EDCs), and pesticides [29].

Advances in nanotechnology and biomolecular engineering have further enhanced the performance of fluorescent sensors, yielding platforms with improved stability, higher quantum yields, and lower detection limits. Commonly employed nanomaterials include carbon dots, graphene oxide, III–V semiconductor quantum dots (e.g., CdS, CdSe, ZnSe) [30], and silicon nanowires (Si NWs) [31].

Overall, fluorescent sensors represent a powerful and cost-effective solution for environmental analysis, enabling real-time monitoring with exceptional sensitivity and selectivity.

Fluorescent sensing platforms have been successfully applied in the detection of several pollutants in water, air, and soil pollution assessment. In this manuscript, an overall review of some of the most interesting results obtained for fluorescent sensors in environmental analyses will be presented. Organic fluorescent sensors will be discussed in detail, reporting their photophysical properties, chemical structures, typical applications, and limitations and giving some insight into their inorganic counterparts in a separate section. As will become evident, organic fluorescent sensors offer several advantages over their inorganic counterparts: they are carbon-based (and thus biodegradable), highly tunable, easy to functionalize, cheaper and easier to synthesize and scale up, biocompatible, and eco-friendly and suitable for green chemistry applications. However, they are typically more affected by photobleaching, sensitive to the pH and polarity, and characterized by narrow Stokes shifts and shorter fluorescence lifetimes (nanoseconds). Conversely, inorganic fluorescent sensors, including III–-V group semiconductor and lanthanide quantum dots, offer high quantum yields, superior photostability, and unique emission properties compared to organic ones, but they possess higher toxicity, limited functionalization, and no biodegradability. The last section will be dedicated to the advancement of portable devices involving fluorescent sensors for real-time monitoring and their industrial perspectives, which may offer a route to more sustainable environmental management, helping in protecting ecosystems and public health.

## 2. Organic Materials

**Figure 1:**
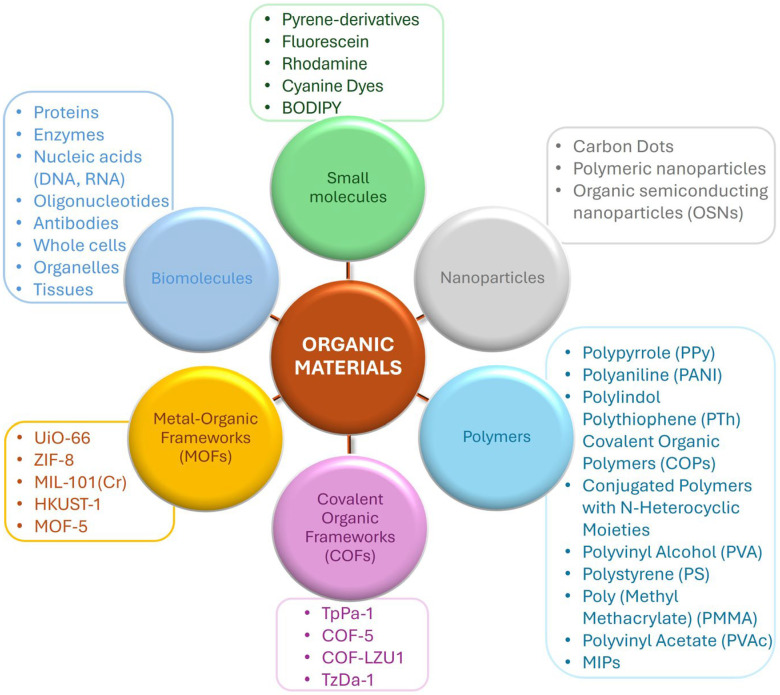
Organic materials mostly used in fluorescent sensors.

Over the last decade, a plethora of organic materials have been synthesized and used to realize organic fluorescent sensors (Figure 1). Due to their high sensitivity, selectivity, low detection limits, and real-time monitoring capabilities, these sensors have emerged as powerful tools for the analysis of various targets.

Depending on the emitting material, organic fluorescent sensors may be broadly classified into six principal categories, although such classification remains somewhat arbitrary, as considerable overlap and interconnection often exist among the different types.

### 2.1. Small Molecule-Based Sensors

Small molecule-based fluorescent sensors use low-molecular-weight organic compounds, typically with a molecular weight of less than 900 Daltons. These molecules are characterized by a relatively simple structure and small size, which allow them to easily penetrate cells and interact with biological targets.

Such characteristics make small molecule-based fluorescent sensors non-invasive and more compatible with live-cell experiments compared with traditional analytical techniques. They also provide higher sensitivity and spatial resolutions compared with electron spin resonance and have the advantage of enabling the real-time monitoring of cell dynamic processes [32]. Small molecule-based fluorescent sensors are also highly complementary to genetically encoded fluorescent protein-based sensors, allowing for the better fine-tuning of their physicochemical properties and a simpler preparation and incubation procedure [33].

**Figure 2:**
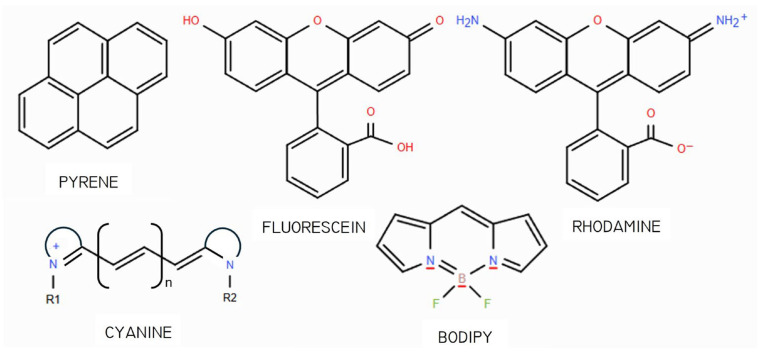
The main organic small molecules used.

Numerous examples of small molecule-based sensors have been reported in the literature in recent years. Rather than giving a complete list, we report here the most investigated molecules (Figure 2).

(1) *Pyrene and its derivatives* are fluorescent probes used in sensors to (*i*) detect reactive oxygen species (ROS) in biological systems (as indicators of oxidative stress and cellular damage) [34]; (*ii*) enhance the electrochemical properties of materials, useful in energy storage applications [34]; and (*iii*) detect various analytes (metals [35], drugs [36], gas molecules [37]). In these sensors, sensing is allowed by changes in pyrene moiety fluorescence intensity. Interesting environmental applications are those in heavy metal detection, with an LOD of 2.9 nM for the detection of Ag^+^ (in DMSO–H_2_O, 1:1 *v*/*v*, HEPES = 50 mM, pH = 7.4) and 4 and 2 ppb for the detection of Hg^+^ and Pb^2+^ (in MeCN–H_2_O, 2:8 *v*/*v*), respectively [35].
(2) *Fluorescein*, a synthetic organic compound with strong fluorescence, is commonly used in glucose sensors for medical diagnostics [38,39] and, along with its derivatives, for modern biochemical and biological studies. Derivatives (Eosin B, Rose Bengal, etc.) are obtained through the inclusion of heavy atoms or the substitution of the hydroxyl groups on the xanthene core in order to tune the fluorescent properties and achieve additional functionalities. The structural modification occurring at the carboxyl group by introducing different groups produces a spirolactam structure that is non-fluorescent. Various factors, like the pH, the temperature, and the presence of other molecules, can cause this modification, followed by quenching. In environmental analysis, fluoresceins have been used to detect different metal ions in aqueous solutions, with LOD values in the nanomolar range: Cu^2+^, noted for the toxic effect of its accumulation in Alzheimer’s or Parkinson’s diseases, was detected down to 6.32 nM [40]; Hg^2+^, which enters the food chain from the environment, was detected down to an LOD of 0.86 nM [41]; and even anions, such as ClO^−^, whose excessive intake can cause tissue damage, liver injury, arthritis, and cardiovascular diseases, was detected with an LOD of 56 pM [42].
(3) *Rhodamine* represents a group of xanthene derivative dyes characterized by fine biocompatibility and near-infrared fluorescence, used in oxygen sensors for environmental monitoring. Structurally similar to fluoresceins, rhodamine sensing is based on the “closing–opening” of the rhodamine derivative’s ring structure, before and after target addition. Coordination between the sensor and target (mainly metal ions [43]) induces spirolactam ring opening and changes the colorimetric and/or fluorescent responses, which are usually absent before the coordination [44]. Optimal LOD values (0.107 µM, corresponding to 6.79 µg/L) have been recently achieved for Cu^2+^, reaching a concentration significantly below the drinking water quality standard of 2.0 mg/L of the World Health Organization (WHO) [43].
(4) *Cyanine dyes* are a family of tetramethylindo(di)-carbocyanines that consist of a polymethine chain containing an odd number of carbon atoms between two tertiary amines, often represented by two aromatic nitrogen-containing heterocycles as charged chromophores, acting as both electron donors and acceptors [45]. Cyanine dyes have been studied widely and are some of the oldest [46] and brightest synthetic fluorophores [47]. They play significant roles in various scientific and technological fields:
  - Biological imaging and microscopy—as fluorescent probes they are widely used for labeling biomolecules such as DNA, RNA, and proteins, allowing researchers to visualize and track biological processes [48];
  - Chemical and biological sensors—due to their sensitive response to changes in their environment, they are employed in the development of sensors for analytes such as ions, H^+^, and biomolecules [48];
  - Photodynamic therapy (PDT)—certain cyanine dyes are used in PDT for cancer treatment, since they generate reactive oxygen species when exposed to light, which can destroy cancer cells [49];
  - Solar cells—cyanine dyes are explored as sensitizers in dye-sensitized solar cells, since they can absorb sunlight and convert it into electrical energy, contributing to the development of renewable energy sources [50].
    While less explored for environmental applications, Galhano et al. proposed silica platforms doped with cyanine derivatives for the detection of divalent metal ions in acetonitrile [51] (Zn^2+^, Cd^2+^, Co^2+^, Ni^2+^, and Hg^2+^), obtaining LOD values of 31 nM and 37 nM, with naked eye detection values of 2.9 ppm and 2.1 ppm, for Hg^2+^ and Cu^2+^ ions, respectively.
(5) *BODIPY (difluoroboron dipyrromethene, or 4,4-difluoro-4-bora-3a,4a-diaza-s-indacene)* represents a class of fluorophores whose structure consists of boron difluoride and dipyrromethene. They possess high photostability and are stable under physiological conditions; moreover, their relative ease of functionalization allows for the extensive synthesis of derivatives, which have been used since the mid-1990s as fluorescent dyes and sensors [52]. A recent interesting application is reported in [53], which describes a fluorometric chemosensor based on mercaptoethanol and BODIPY constructed to detect dicofol, an organochlorine pesticide that is moderately toxic to humans. The chemosensor displayed turn-off fluorescence behavior under dicofol, with a detection limit of 200 ppb. In another study [54], a water-soluble near-infrared sensor based on aza-BODIPY was developed for the dual determination of Cd^2+^ in environmental and biological media. The sensor exhibited a color change from colorless to green along with a fluorescence enhancement in the near-infrared (NIR) region via photoinduced electron transfer (PET) after complexation with Cd^2+^, with an LOD of 2.8 ppb.

In addition to the conventional sensors previously described, it is worth mentioning those based on bicolor signal shifts of small benzo[4,5]imidazo[2,1-b]quinones. In particular, a recent article has introduced monocolor and bicolor fluorescent sensors based on crown ether–fluorophore conjugates, specifically tailored to Ba^2+^ detection, in both solution and solid–gas interfaces [55]. These supramolecular systems exhibit turn-on fluorescence and wavelength shifts upon ion binding, offering a modular and highly selective approach for barium tagging in rare event physics, such as neutrinoless double beta decay. In another study [56], an analogous turn-on probe has been used for the selective and sensitive detection of Cu^2+^ ions by exploiting the blue shift in the emission maxima and enhanced fluorescence achieved after binding with copper and due to reduced non-radiative decay.

### 2.2. Nanoparticle-Based Sensors

Organic nanoparticles have emerged as a revolutionary class of materials in sensor technology. These nanoscale particles exhibit unique physical, chemical, and optical properties that make them highly suitable for various sensing applications. Their high surface area to volume ratios, biocompatibility, tunable properties, and ease of functionalization have encouraged their use for the detection of a wide range of targets, including ions, molecules, and biological entities.

The versatility of organic nanoparticles allows for their integration into various sensor platforms, including optical, electrochemical, and biosensors. This adaptability, coupled with their environmentally friendly and cost-effective synthesis, has driven significant research and development efforts aimed at harnessing their potential in real-world applications. From medical diagnostics to environmental monitoring, organic nanoparticles are paving the way for the next generation of high-performance sensors, offering unprecedented accuracy and reliability. However, one of the key challenges associated with their synthesis and practical deployment is the tendency of nanoparticles to aggregate, which can significantly reduce their active surface area and alter their physicochemical behavior, ultimately compromising sensor performance. This phenomenon arises from interparticle forces such as van der Waals interactions and electrostatic attractions, leading to the formation of larger clusters that reduce the surface area and mask active sites. To mitigate the effects of aggregation, strategies such as surface functionalization with stabilizing ligands, the use of core–shell architectures, and controlled synthesis conditions are employed. These approaches help to maintain colloidal stability and preserve the nanoparticles’ performance in sensing environments.

Carbon quantum dots (CQDs) represent one of the most prominent types of organic nanoparticles used in sensors. Known for their exceptional fluorescence and photostability, CQDs have been extensively utilized in the detection of heavy metal ions, environmental pollutants, and biomolecules. Other organic nanoparticles, such as polymeric nanoparticles and organic semiconducting nanoparticles (OSNs), have also shown great promise in enhancing the sensitivity and specificity of sensors.

**Figure 3:**
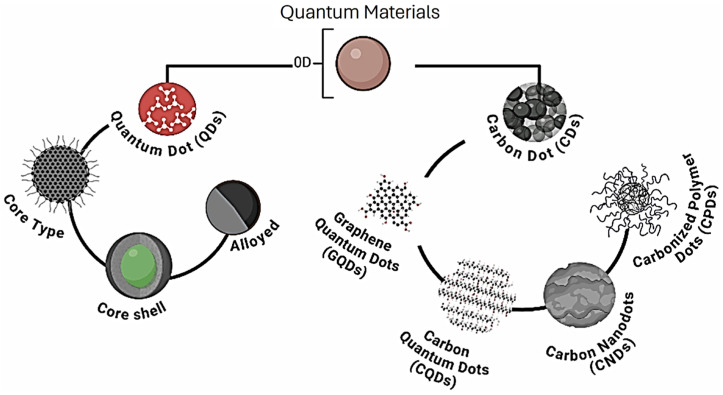
Quantum materials: differences between quantum and carbon dots [61]. Reproduced with permission from [61]. Copyright 2025, Elsevier.

**Figure 4:**
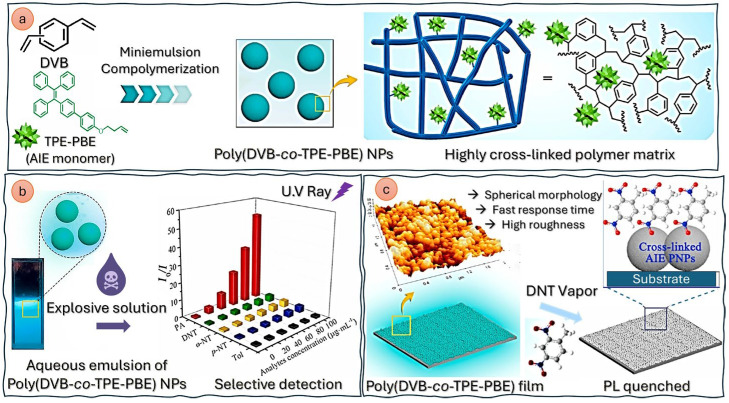
Diagram of highly crosslinked AIE polymer nanoparticles employed in explosive detection. (**a**) Preparation of highly crosslinked poly(DVB-*co*-TPE-PBE) NPs via mini-emulsion copolymerization; (**b**) selective detection of picric acid (PA) in aqueous media; and (**c**) volatile explosive detection by poly(DVB-*co*-TPE-PBE) film [73]. Reproduced with permission from [73]. Copyright 2021, Elsevier.

(1) *Carbon quantum dots (CQDs)* are a fascinating class of carbon-based nanomaterials known for their low toxicity, high biocompatibility, ease of synthesis (even from biomass and waste), and broad possibilities for surface modification or heteroatom doping [57]. These zero-dimensional nanoparticles have garnered significant attention for their potential applications in various fields, including biosensing for the detection of metal ions and (bio)molecules [58], bioimaging, and drug delivery [59]. As shown in Figure 3, belonging to the group of carbon dots, CQDs substantially differ from quantum dots (QDs): the latter have the same structure and atomic composition as bulk materials, while the former are quasi-spherical nanoparticles with both amorphous and crystalline cores, composed of graphitic or turbostratic carbon with an sp^2^ configuration, and may also consist of graphene or graphene oxide sheets fused or arranged through sp^3^-hybridized carbon atoms [60]. Due to the abundance of functional groups on their surfaces, CQDs possess high water solubility and provide active sites for functionalization, thus enhancing their versatility and applicability in different fields.
  The use of CQDs for environmental applications in the sensor and biosensor areas is widely reported in the literature. Most CQD-based sensors focuses on heavy metal detection, and, among them, mercury is the most frequently detected, with an LOD of 0.2 nM, useful for effective water quality control [62]. Meanwhile, most CQD-based biosensors targeting environmental pollutants focus on pesticides, such as diazinon [63], glyphosate [63], fenitrothion [64], malathion [65], and chlorpyrifos [65], with LODs ranging from 0.36 to 8.2 nM.
  An interesting environmental application is microplastic detection. In [66], N and Cl co-doped carbon quantum dots were synthesized and used to combine fluorescence and Rayleigh scattering, enabling the detection of polystyrene microplastics with three different particle sizes, with an LOD of 0.4 mg/L. Due to their remarkable photostability, CQDs are particularly suitable for real-time monitoring [67].
(2) *Polymeric nanoparticles* are nanoscale particles composed of polymers, which have gained significant attention in various fields due to their numerous properties: they are biocompatible—and thus suitable for medical and biological applications—and can be designed in various shapes (spheres, rods, or capsules) depending on the intended application. Moreover, they can be engineered to minimize toxicity and immune responses and can be easily modified with various functional groups, targeting ligands, or therapeutic agents, enhancing their specificity and effectiveness. Examples of polymeric nanoparticles are polyethylene glycol (PEG) nanoparticles, polylactide-co-glycolide (PLGA) nanoparticles, or conductive polymer nanocomposites (CPNs), i.e., a combination of conductive polymers with materials like graphene, carbon nanotubes, and metal nanoparticles. Although most of the literature on polymeric nanoparticles centers on their use in drug delivery systems [68,69], imaging/diagnostics [70], gene therapy [71], and cancer treatment [69], compelling studies also demonstrate their effective utilization as chemical sensors [72]. In [73], a highly crosslinked polymer matrix, obtained by the mini-emulsion copolymerization of divinylbenzene (DVB) with an aggregation-induced emission (AIE) monomer composed of (4′-tetraphenyletheyl)pheny-3-butenylether (TPE-PBE), is designed for the detection of explosives based on the quenching of the polymer matrix (both in liquid and film states) observed after contact with the target (in solution or as a vapor) (Figure 4). The sensor showed very good sensitivity for the highly explosive picric acid, with an LOD value of 5.43 μM (or 1.24 ppm).
  Another notable application lies in the detection of Fe^2+^ and Fe^3+^ through a chemosensor based on fluorescent polymer nanoparticles bearing rhodamine B ethylenediamine acrylate, obtained by semicontinuous emulsion polymerization [74]. The color of the aqueous dispersion of polymer nanoparticles changed from violet to red–pink immediately after adding iron ions, with LOD values of 2.63 and 2.5 µM for Fe^2+^ and Fe^3+^, respectively.
(3) *Organic semiconducting nanoparticles (OSNs)* are a class of nanomaterials derived from organic semiconductors, which are composed primarily of carbon and hydrogen atoms. They exhibit unique electronic and optical properties, which make them highly suitable for various applications, including biosensing, imaging, and optoelectronics [75,76]. Examples of OSNs are (*i*) poly(3-hexylthiophene) (P3HT) nanoparticles, used for applications in organic photovoltaics and biosensors [77,78]; (*ii*) polydiacetylene (PDA) nanoparticles, known for their colorimetric and fluorescence properties and useful in biosensing and environmental monitoring [79]; (*iii*) polyfluorene nanoparticles, used in optoelectronic devices, such as organic light-emitting diodes (OLEDs) and organic field-effect transistors (OFETs) [80]; and (*iv*) poly(9,9-dioctylfluorene-co-bithiophene) (F8T2) nanoparticles, employed in organic solar cells and biosensors due to their excellent charge transport properties [81].

**Figure 5:**
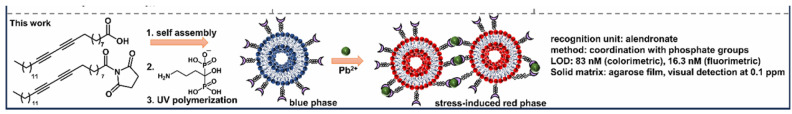
Colorimetric sensor for lead ions based on PCDA-Alen [82]. Reproduced with permission from [82]. Copyright 2024, American Chemical Society.

**Figure 6:**
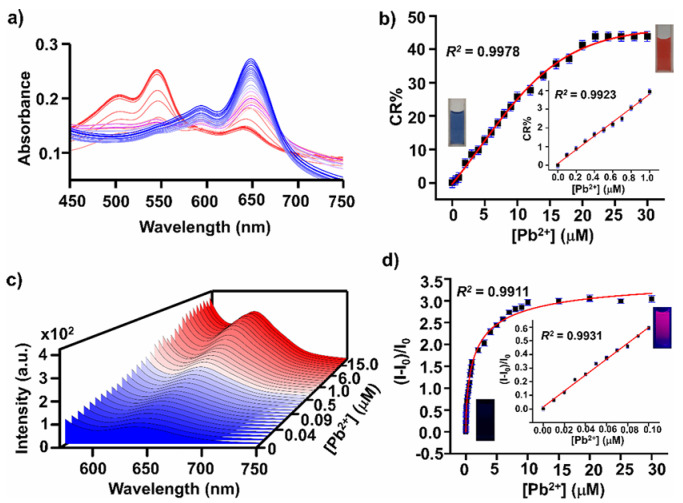
(**a**) Response of PCDA-Alen liposomes upon the addition of Pb^2+^ ions in UV−vis spectroscopy (blue lines: before Pb addition; red lines: after Pb addition). (**b**) Colorimetric response of PCDA-Alen liposomes upon the addition of lead ions (0−20 μM) [inset: colorimetric response of PCDA-Alen liposomes at lower concentrations of Pb^2+^ ions (0−1 μM)]. (**c**) Fluorimetric response of PCDA-Alen liposomes upon the addition of Pb^2+^ ions. (**d**). Increase in relative fluorescence output upon the addition of Pb^2+^ ions (0−30 μM) [inset: linear correlation between the relative increase in fluorescence intensity and the concentration of Pb^2+^ ions (0.01−0.1 μM)] [82]. Reproduced with permission from [82]. Copyright 2024, American Chemical Society.

In [82], a sensitive and selective dual-mode sensor based on PDA, utilizing the phosphate groups of the alendronate unit as a quick recognition element for Pb^2+^ ions in both solution and solid phases, was realized. Specifically, 10,12-pentacosadiynic acid (PCDA)-derived vesicles were modified with alendronate units (PCDA-Alen) to enable the specific recognition of lead ions in the presence of other metal ions and possible interfering species. Upon UV irradiation (254 nm), PCDA-Alen displayed a characteristic blue color that, after interaction with lead ions, exhibited a characteristic color transition from blue to red, accompanied by a gradual increase in fluorescence output at a λ_max_ value of 642 nm (Figure 5). The sensor was indifferent to a variety of cations or anions and displayed, toward Pb^2+^, LOD values of 16.8 ppb in UV−vis spectroscopy and 3.1 ppb in fluorescence spectroscopy (Figure 6).

In [83], a paper-based naked-eye colorimetric sensor array comprising 10,12-tricosadiynoic acid (TCDA) and N-1-hexadecyl imidazole derivatives was prepared to sense 12 different volatile organic compounds (VOCs). The TCDA-clay-N-1-hexadecyl imidazole sensor could detect a minimum concentration of 0.02% for toluene and benzene in 6 min, whereas it could detect a concentration of 0.08% for THF and 0.04% for benzene in 80 s.

### 2.3. Polymer-Based Sensors

Polymer-based sensors are an innovative and rapidly evolving field due to their versatility, flexibility, and cost-effectiveness. They leverage the unique properties of polymers to detect various physical, chemical, and biological stimuli, offering the possibility of creating highly sensitive and versatile devices for a wide range of applications, from environmental monitoring to healthcare diagnostics [84]. Recent studies have highlighted the progress and challenges in polymer-based sensors. For instance, a review published in *Polymers* discusses the current trends and future prospects of these sensors, emphasizing their rapid detection capabilities, small size, high sensitivity, and suitability for various atmospheric conditions [85]. Another review in *Chemosensors* provides an overview of the trends and challenges in sensor research, illustrating the versatility of polymers in sensing applications [86]. According to the literature cited in this review, the detection limits of general organic sensors and specifically conductive polymers such as PPy, PANI, and inorganic alternatives are generally comparable. Several studies highlight PANI as a highly sensitive platform [87]. Nevertheless, inorganic materials, particularly 2D MoS_2_, have demonstrated exceptional detection limits, reaching the femtomolar range [88]. For optical sensors targeting biomolecules or ions, response times are not frequently reported, as they often correspond to the measurement duration. In the case of gas sensors, the response time is influenced by multiple factors, including the type of gas, its concentration, the temperature, and other experimental conditions. Typically, the response time increases with decreasing analyte concentrations, ranging from sub-second responses at ppm levels to tens of minutes at concentrations below hundreds of ppm [89]. Overall, while inorganic alternatives can surpass conducting polymers in certain aspects of sensing performance, PPy and PANI remain attractive due to their low costs, ease of functionalization, and versatility across different sensing environments. In many practical applications, their performance is adequate, and the optimal choice depends on balancing cost-effective fabrication, material stability, and the specific requirements of the target analyte.

These advancements underscore the potential of polymer-based sensors in various fields, making them a crucial component of modern sensing technologies. There are two different types of polymers: conducting and non-conducting.

#### 2.3.1. Conducting Polymers

**Figure 7:**
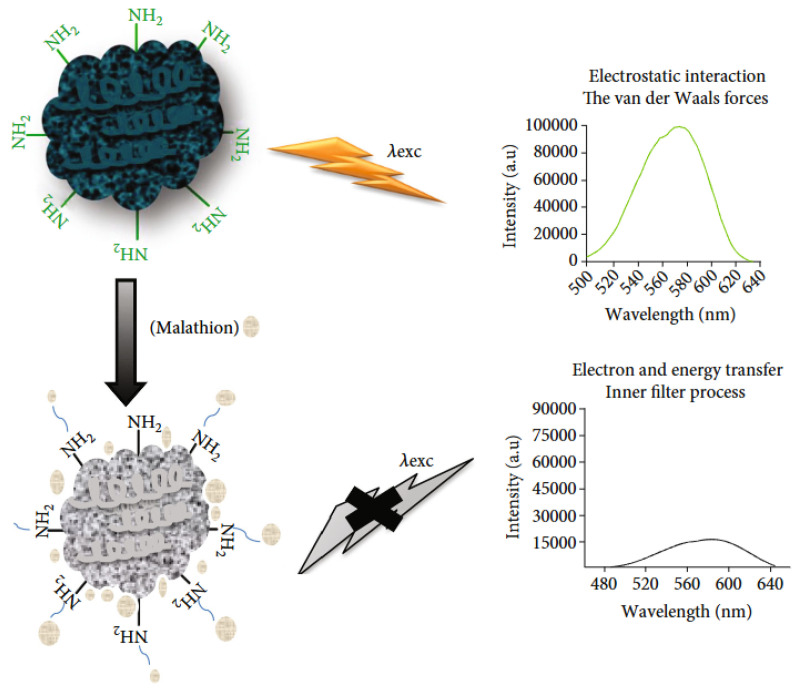
Schematic illustration of malathion (MA) detection using Ag-ZnO/PANI NC fluorescence sensor [92]. Reproduced with permission from [92]. Copyright 2022, John Wiley and Sons. Open access.

**Figure 8:**
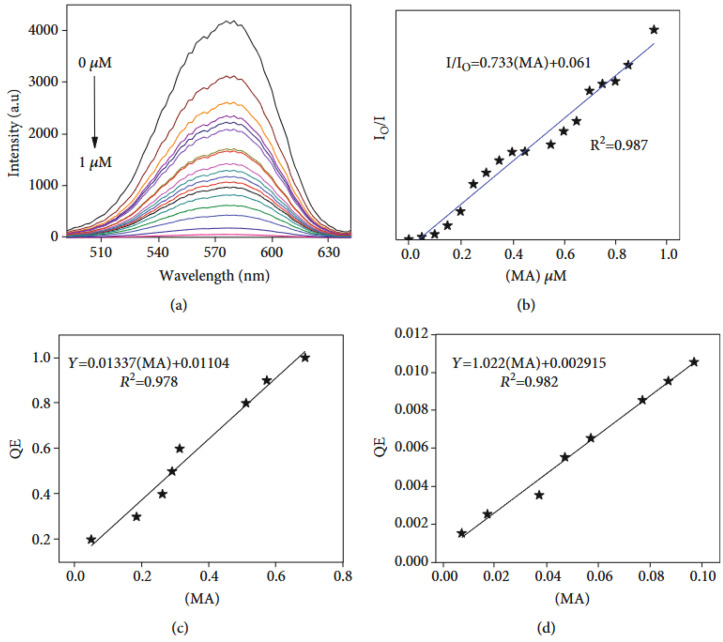
(**a**) Fluorescence quenching of Ag-ZnO/PANI NC with increasing concentrations of malathion (MA); (**b**) linear relationship plot representing I_o_/I and concentration of MA, where *I_o_* and *I* are the fluorescence intensities of the Ag-ZnO/PANI nanocomposite in the absence and presence of MA; (**c**) QE versus MA concentration from 0.1 to 0.8 μM and (**d**) from 0.01 to 0.1 μM at pH 7 and *λ*_ex_ = 380 nm [92]. Reproduced with permission from [92]. Copyright 2022, John Wiley and Sons. Open access.

**Figure 9:**
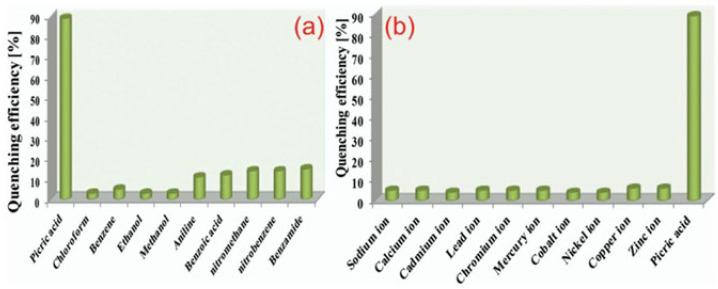
(**a**) Selectivity of a PIN/CdS nanocomposite in the presence of organic compounds and (**b**) selectivity of a PIN/CdS nanocomposite in the presence of metal ions [96]. Reproduced with permission from [96]. Copyright 2017, Royal Society of Chemistry.

**Figure 10:**
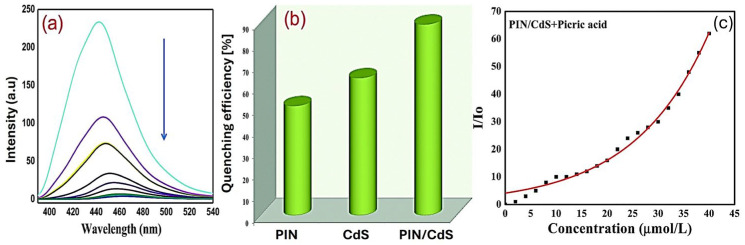
(**a**) Fluorescence spectra of PIN/CdS nanocomposite with increasing concentrations of picric acid (the arrow indicates the fluorescence intensity decrease, by increasing the concentration of picric acid); (**b**) quenching efficiency of PIN, CdS, and the PIN/CdS nanocomposite; (**c**) Stern–Volmer plot of the PIN/CdS nanocomposite in the presence of picric acid (Data are reported as black dot fitted as red line) [96]. Reproduced with permission from [96]. Copyright 2017, Royal Society of Chemistry.

**Figure 11:**
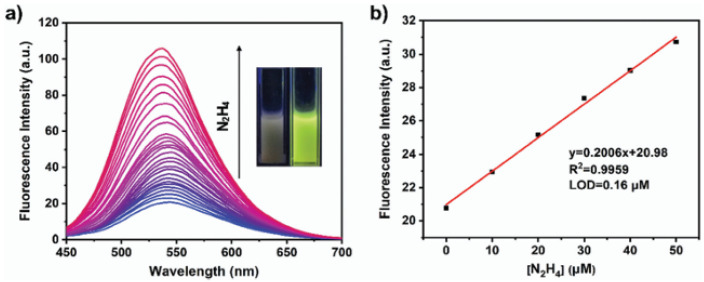
(**a**) Fluorescence emission spectra of COP-Ta dispersed in ethanol after adding different concentrations of hydrazine solution (from bottom to top: 0, 0.01, 0.02, 0.03, 0.04, 0.05, 0.1, 0.15, 0.2, 0.5, 0.8, 1.0, 2.0, 4.0, 6.0, 10.0, 15.0, 20.0, 30.0, 40.0, 50.0, 60.0, 80.0, 100.0, 125.0, 150.0, 175.0, and 200.0 mM) (lex = 365 nm). Inset: A photograph of the fluorescence emission change (under a UV lamp with lex = 365 nm) of COP-Ta in ethanol upon the addition of 200 mM of hydrazine. The arrow indicates how fluorescence intensity increases, by increasing the concentration of hydrazine. (**b**) The linear relationship between the fluorescence emission intensity of a COP-Ta suspension at 543 nm and the concentration of hydrazine [103]. Reproduced with permission from [103]. Copyright 2022, Royal Society of Chemistry.

(1) *Polypyrrole (PPy)* is a conductive polymer known for its excellent electrical properties and environmental stability, which make it suitable for various optical sensing applications. PPy is widely used in chemical and biological sensors. Its ability to undergo reversible redox reactions makes it suitable for detecting gases, ions, and biomolecules [84] and metals with a negative impact on the environment [90].
(2) *Polyaniline (PANI)* is valued for its tunable conductivity and ease of synthesis. It is used in sensors for detecting pH changes, gases, and humidity. Its conductive properties can be adjusted by doping with different acids [91], while, in the form of a nanocomposite based on electrostatic interactions between polyaniline and Ag-ZnO, it has been used for the detection of organophosphorous pesticides [92] (Figure 7 and Figure 8). Polypyrrole (PPy) and polyaniline (PANI) exhibit poor electrochemical stability when used as pure polymers [93]. However, researchers have been working to solve this issue and, for example, hydrogels based on these polymers have shown significantly improved stability. According to the literature, polyaniline- and polypyrrole-based hydrogels enhance performance by up to two orders of magnitude and maintain stability over thousands of working cycles [94].

(3) *Polyindol (PIN)* is a polymer of an indole monomer that has a fused aromatic molecular structure consisting of a five-membered nitrogen-containing pyrrole ring and a six-membered benzene ring. In comparison to other conducting polymers, such as PANI, PIN shows relatively slow hydrolytic degradation, improved thermal stability, and excellent photoluminescent properties [95]. A noteworthy, albeit earlier, application of PIN is that reported by Faraz et al. [96] for the detection of picric acid. The fluorescence sensor, obtained from the in situ chemical oxidative polymerization of polyindole with CdS nanoparticles, worked based on a static/dynamic quenching mechanism. The comparison of the fluorescence intensities with those of diverse metal ions, such as Li^+^, Ca^2+^, Cd^2+^, Pb^2+^, Cr^2+^, Hg^2+^, Co^2+^, Ni^2+^, Cu^2+^, and Zn^2+^, confirmed the selectivity of the PIN/CdS nanocomposite for picric acid (Figure 9), showing a Stern–Volmer constant (*K*_sv_) value of 30 × 10^3^ M^−1^ (Figure 10) [96].

(4) *Polythiophene (PTh)* is used with its derivatives for their excellent electrical properties and stability. PTh-based sensors are employed in applications such as gas detection and biosensing [97]. One of its derivatives (PEDOT), combined with polystyrene sulfonate, is particularly popular in flexible and wearable sensors [98], while malonic acid derivatives (PTMA) have been used for the detection of alkaloids [99].
(5) *Covalent organic polymers (COPs)* are a class of materials characterized by highly stable, covalently bonded structures. These polymers are composed of organic building blocks linked together through strong covalent bonds, forming extended networks. Their high porosity and tunable properties make them suitable for a wide range of applications, such as gas storage [100], catalysis [101], and sensing [102], for the detection of heavy metal ions, explosives, and biological molecules. Due to their high thermal stability, easily adjustable functions, amplified responses, and superior sensitivity to analytes (compared with low-molar-mass congeners), COPs are promising candidates for fluorescent sensors. In [103], for instance, a COP containing ynone reaction sites (namely, COP-Ta) was used for detecting hydrazine (Figure 11).

(6) *Conjugated polymers with N-heterocyclic moieties* are a class of polymers that incorporate N-heterocyclic moieties into their conjugated backbones. This enhances the electronic properties, such as conductivity and fluorescence, making them highly effective in detecting various analytes, such as environmental pollutants [104] and bioactive compounds [105]. This performance is further amplified by the molecular wire effect, a phenomenon first described by Zhou and Swager in the early 1990s [106]. It refers to the efficient transport of electronic excitations, or charge carriers, along the polymer backbone, facilitated by the delocalization of π-electrons across the conjugated system. This extended conjugation creates a quasi-one-dimensional pathway that enables rapid energy migration, allowing localized interactions, such as analyte binding or charge trapping, to influence the entire polymer chain. As a result, materials exhibiting this effect demonstrate enhanced sensitivity and signal amplification, particularly in applications such as fluorescent chemosensing and organic electronics. Examples of conjugated polymers with N-heterocyclic moieties are poly(2,5-bis(3-hexylthiophen-2-yl)thiazolo[5,4-d]thiazole) (PBTTT), whose thiazole units improve charge transport properties (useful in organic field-effect transistors (OFETs) and photovoltaic devices) [107]; poly(3-hexylthiophene) (P3HT), whose pyridine units enhance electron-accepting properties (useful for applications in organic solar cells and sensors) [108]; and poly(2,7-carbazole) derivatives, known for their high thermal stability and excellent optoelectronic properties, which make them useful for light-emitting diodes, photovoltaic devices [109], and optical sensors [110].

According to the doping level and type, composite formation (e.g., with nanoparticles or carbon materials), environmental conditions (pH, temperature, redox cycles), and morphology, the reusability of conducting polymers can vary from moderate (as for PTh, which has limited reusability under oxidative conditions [111]) to high (such as in the case of PANI and COPs [112]), with some examples of polymers showing promising, but still less explored, reusability (such as PIN [95]), or variable, but still under active research, reusability (such as conjugated polymers with N-heterocyclic moieties, which can greatly differ depending on the heterocycle and backbone structure).

#### 2.3.2. Non-Conducting Polymers

**Figure 12:**
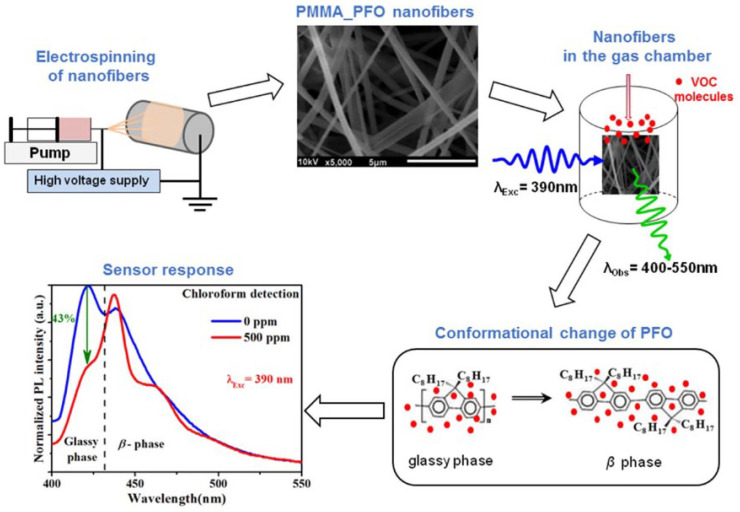
Schematic view of PMMA_PFO nanofibers obtained by electrospinning and used as VOC gas sensors. The conformational change in PFO in the presence of volatiles results in fluorescence quenching and, consequently, in chloroform sensing [117]. The vertical dashed line indicates the boundary between the glassy- and the β-phase. Reproduced with permission from [117]. Copyright 2017, John Wiley and Sons.

**Figure 13:**
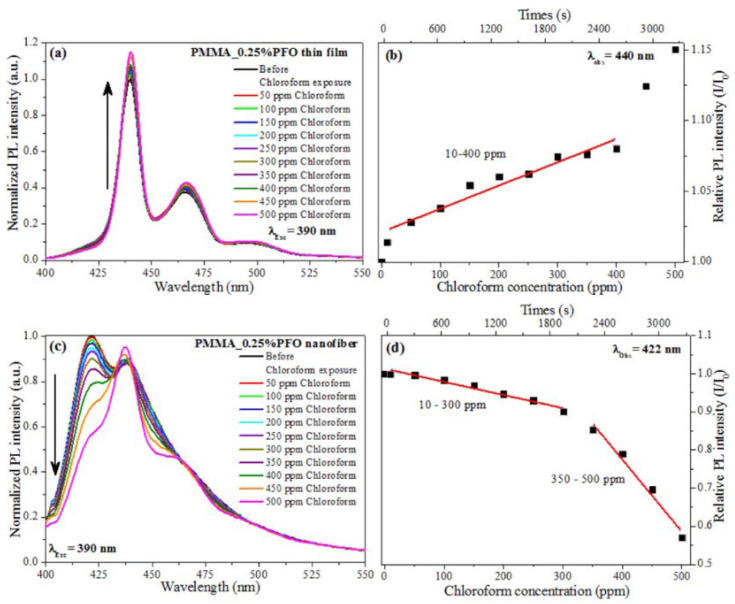
PL spectra of (**a**) PMMA_0.25%PFO thin film and (**c**) PMMA_0.25%PFO nanofiber, both excited at 390 nm, before and after exposure to chloroform vapor (concentrations shown in the captions). (**b**,**d**) show the dependence of the emission intensity as a function of the chloroform concentration for the PMMA_0.25%PFO thin film (monitored at 440 nm) and PMMA_0.25%PFO nanofiber (monitored at 420 nm), respectively [117]. Reproduced with permission from [117]. Copyright 2017, John Wiley and Sons.

(1) *Polyvinyl alcohol (PVA)* is a water-soluble polymer used in sensors for its excellent film-forming properties and biocompatibility. It is often used in combination with other materials to enhance the sensitivity and selectivity of sensors in detecting humidity, gases, biomolecules [86], and metals [113].
(2) *Polystyrene (PS)* is a polymer with excellent hydrocarbon resistance, a high degree of dimensional stability, and superior mechanical performance at high temperatures. As nanobeads, microspheres, ultrathin films, or in association with other polymers (such as polyvinyl chloride, PVC), it has been used for the detection of alcohols, Fe^3+^, and chloroform [84], while, in association with fluorophores (like naphthalimide), it has been used for sensing heavy metals [114].
(3) *Poly (methyl methacrylate) (PMMA)* is a synthetic polymer prepared from a methyl methacrylate monomer with the use of different polymerization methods. It is widely used for the sensing of pH [115] and gases such as NO_2_ [116] and, interestingly (as nanofibers), to detect volatile organic compounds (VOCs) [117]. In this study, electrospun nanofibers composed of PMMA doped with small amounts of polyfluorene (PFO) were used to realize a sensor, showing fluorescence quenching in the presence of various VOCs, particularly chloroform, with detection limits as low as 15.4 ppm. The sensing mechanism was attributed to conformational transitions of PFO from the glassy phase to the β-phase, induced by interactions with VOC molecules, resulting in changes in its photophysical behavior (Figure 12 and Figure 13).

(4) *Polyvinyl acetate (PVAc*) is prepared by the polymerization of a vinyl acetate monomer; its reactivity depends on the triple-bond electronic density and the π-bond energy. It has been successfully employed for ammonia sensing [118] or as a filter (integrated with a complementary metal-oxide semiconductor imaging sensor) for the realization of a portable biochemical sensing device [119]. Moreover, it was recently used as randomly oriented nanofibers to realize a sensor with a surface-enhanced fluorescence factor of 1170 and an LOD as low as 7.24 fM for rhodamine 6G (used as a probe) [120].

A separate subclass of polymer-based sensors is represented by MIPs, a group of polymeric materials engineered to possess selective recognition sites that are complementary in shape, size, and chemical functionality to a target analyte and mimic the binding specificity of biological receptors such as antibodies. MIPs are synthesized by polymerizing functional monomers and crosslinkers in the presence of a template. Upon removal of the template, the polymer retains structurally and functionally complementary cavities, which act as selective binding sites.

Operating primarily through molecular recognition and selective binding, rather than functioning through intrinsic electrical properties, MIPs do not fall neatly into the conventional dichotomy of conductive versus non-conductive polymers. Since their discovery in the 1970s by Günter Wulff [121], they have been extensively used in chromatography, catalysis, and various sensing applications. For detailed insights into MIPs, readers may consult the considerable volume of domain-specific publications published over the last 50 years. MIPs do not typically exhibit useful intrinsic fluorescence; some highly crosslinked aromatic polymers may show weak background autofluorescence [122,123] but this is typically not suitable for sensing applications [124]. Fluorescent MIPs almost always rely on fluorescent monomers, crosslinkers, or embedded fluorescent species [125].

### 2.4. Covalent Organic Framework (COF)-Based Sensors

**Figure 14:**
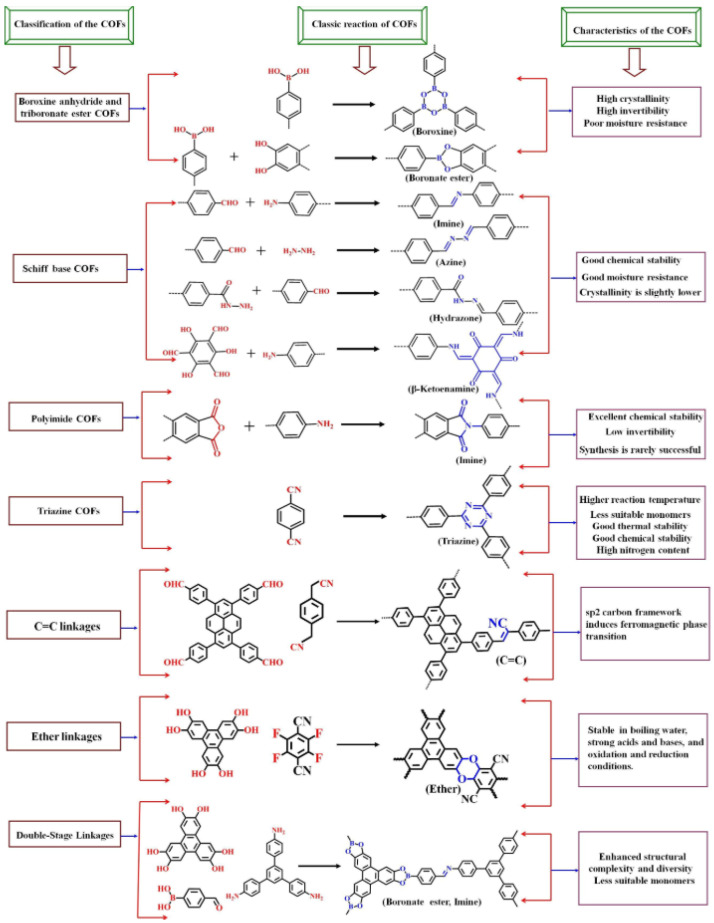
Classification, classical reaction, and characteristics of COFs [126]. Reproduced with permission from [126]. Copyright 2021, Elsevier.

COFs are a class of porous, crystalline materials formed by the covalent bonding of organic building blocks (Figure 14) [126].

These materials appeared for the first time in 2005, thanks to the pioneering work conducted by Adrien P. Côté et al. [127], who described the synthesis of 2D COFs. Designed both in two- and three-dimensional structures, COFs are known for their high surface area, tunable porosity, and exceptional stability, but with substantial differences compared to the above-mentioned COPs, which have a longer history, rooted in the broader field of polymer science. The differences between them mainly concern the structure and porosity: on one hand, COFs are crystalline and have a well-defined, periodic structure, whereas COPs are typically amorphous or semi-crystalline; on the other hand, COFs are highly porous with uniform pore sizes, while COPs may have less defined porosity. Compared to other fluorescent detection probes, such as metal organic frameworks (MOFs), which are discussed below, the covalent bonds that form the backbones of COFs are more robust than the metal–ligand bonds in MOFs, providing them with strong luminescent properties and broadening their detection applications [128]. In contrast to amorphous polymers employed as MIPs, covalent organic frameworks (COFs) offer a significantly larger surface area, which contributes to lower detection limits. More broadly, crystalline polymers can mitigate the random quenching pathways that often prevail in amorphous systems [129]. The ordered domains present in conjugated crystalline polymers also facilitate more efficient exciton migration, thereby enabling signal amplification, whereas amorphous counterparts tend to localize excitons, resulting in diminished fluorescence yields [130]. In addition, the presence of crystalline regions generally enhances both thermal and mechanical stability [131].

COFs can be used in a wide range of applications, including gas storage, catalysis, drug delivery, and environmental pollutant detection [132,133]. In environmental optical sensing applications, they are mainly used for detecting heavy metal ions (like Pb and Hg) and explosives (such as 2,4,6-trinitrotoluene (TNT), dinitrotoluene (DNT), and hexahydro-1,3,5-trinitro-1,3,5-triazine (RDX)), thanks to their high surface areas and tunable properties, which enhance their sensitivity and selectivity.

**Figure 15:**
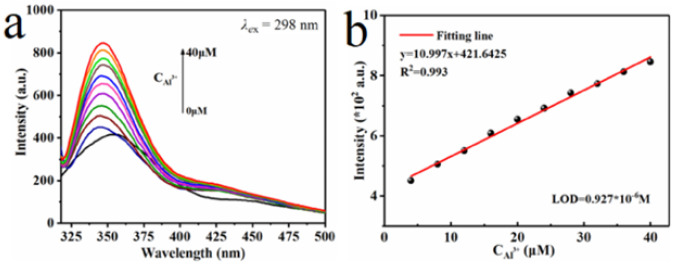
Fluorescence titration spectra (**a**) and calibration curve (**b**) of COF-DHTA (0.05 mg/mL) in DMF upon the incremental addition of Al^3+^ ions [134]. Reproduced with permission from [134]. Copyright 2021, Elsevier.

A particularly compelling example of heavy metal detection is described by Xiu et al. [134], who developed a COF-DHTA using tetraphenylethene, with exceptional thermal stability, crystallinity, and selectivity towards Al^3+^ (LOD 0.93 µmol/L) (Figure 15).

**Figure 16:**
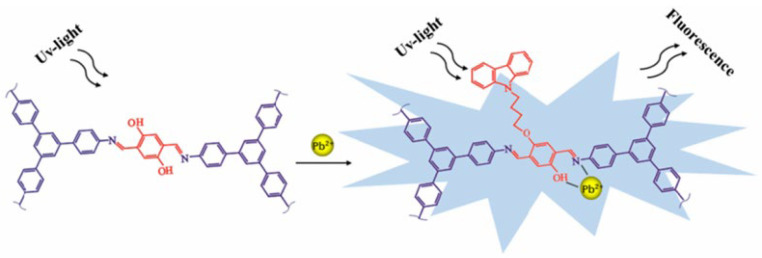
Proposed binding mode of COF-CB with Pb^2+^ ions. Reproduced with permission from [135]. Copyright 2021, Springer Nature.

**Figure 17:**
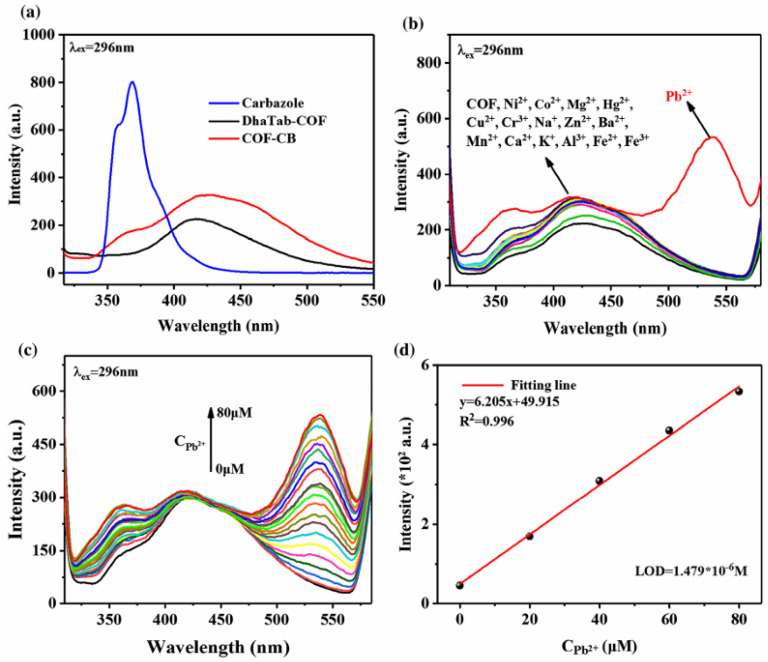
(**a**) Fluorescence emission spectra of carbazole, COFDhaTab, and COF-CB in DMF; (**b**) fluorescence spectra after adding 16 metal ions (20 mM, 20 µL) to COF-CB; (**c**) fluorescence spectra changes of COF-CB in the presence of Pb^2+^ with different concentrations; (**d**) linear relationship between the concentration of Pb^2+^ and the fluorescence intensity [135]. Reproduced with permission from [135]. Copyright 2021, Springer Nature.

Another example is described by Zhang et al. [135], where a new carbazole-grafted covalent organic framework (COF-CB) was realized as an enhanced fluorescent chemosensor for the recognition and detection of Pb^2+^ ions, with an LOD of 1.48 µM. Figure 16 describes the binding mechanism proposed for this sensor [135], while the sensing performance is shown in Figure 17.

**Figure 18:**
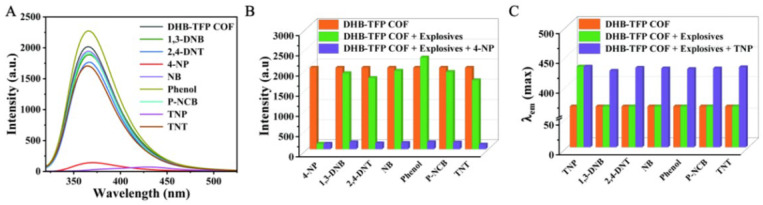
(**A**) Fluorescence emission spectra of DHB-TFP COF in ethanol when adding different explosives. (**B**) Fluorescence intensities of DHB-TFP COF in ethanol upon the addition of different explosives and 4-NP. (**C**) Maximum fluorescence peaks of DHB-TFP COF in ethanol upon the addition of different explosives and TNP [136]. Reproduced with permission [136]. Copyright 2024, Elsevier.

In [136], a novel hydroxyl-rich covalent organic framework (DHB-TFP COF) is proposed to detect 4-nitrophenol (4-NP) and trinitrophenol (TNP), with an LOD of 0.40 μmol/L for 4-NP and 11.15 μmol/L for TNP (Figure 18).

Other popular COFs used for optical sensors are as follows.

(1) TpPa-1 is composed of 1,3,5-triformylphloroglucinol (Tp) and p-phenylenediamine (Pa), known for its excellent chemical stability and high fluorescence efficiency and widely used in sensing applications, including the detection of metal ions and explosives [137].
(2) COF-5 is constructed from benzene-1,4-diboronic acid and hexahydroxytriphenylene. It exhibits a three-dimensional structure with high thermal stability and is used in applications such as gas adsorption and catalysis, as well as fluorescent sensing [138].
(3) COF-LZU1 is synthesized from 1,3,5-triformylbenzene and 1,4-phenylenediamine. It has a two-dimensional structure and is known for its high surface area and stability, making it suitable for catalysis and gas storage [139] but also for detecting various analytes via fluorescent sensors [138].
(4) TzDa-1 is derived from 1,3,5-triformylphloroglucinol (Tz) and 2,5-diaminobenzene (Da). It is known for its high fluorescence quantum yield and is used in sensing applications for detecting environmental pollutants [140].
(5) COF-300 is constructed from tetra(4-dihydroxyborylphenyl)methane and 1,4-benzenediboronic acid. It features a three-dimensional structure with high porosity and is used in applications such as gas storage, separation, catalysis [139], and fluorescent sensing [138].

Although substantial advances have been made in recent years, significant challenges remain unaddressed. Most of the investigated COF sensors, for instance, enable fluorescence quenching, while fluorescence enhancement against dark backgrounds is more reliable for sensing purposes. Moreover, most of the COFs are water-insoluble and require dispersion in various solvents, with limitations in terms of sustainability and the possibility of solid-state fluorescence detection [141].

### 2.5. Metal–Organic Framework (MOF)-Based Sensors

**Figure 19:**
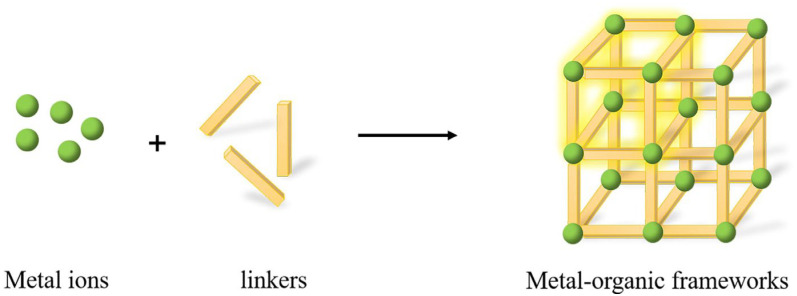
Scheme for the preparation of an MOF, showing metals mixed with linkers. Reproduced with permission from [142]. Copyright 2020, Elsevier.

MOFs are a class of crystal hybrids assembled from inorganic metal ions, or clusters, and organic ligands, which create a porous, crystalline framework forming one-, two-, or three-dimensional structures (Figure 19) [142].

**Figure 20:**
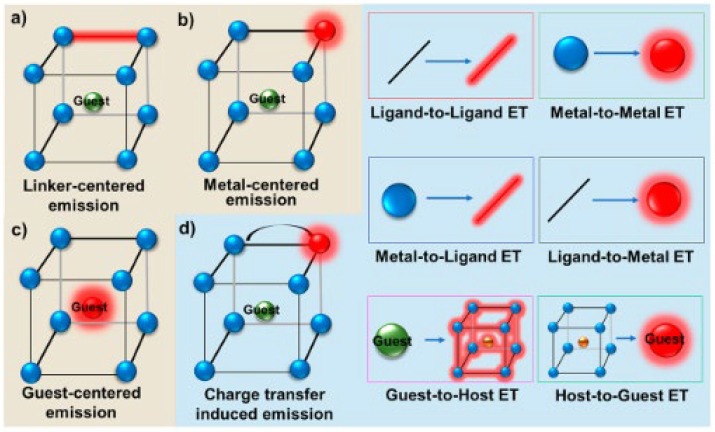
Scheme of the possible fluorescence origins and the different energy transfer processes in MOFs. Reproduced with permission from [143]. Copyright 2022, American Chemical Society. Open access. (**a**) Emission from the organic linker; (**b**) Emission from the metal node; (**c**) Emission from the guest molecule; (**d**) Emission induced by charge transfer.

Known for their high surface areas, tunable pore sizes, and ability to incorporate various functional groups, MOFs are useful in a variety of applications, such as gas storage, catalysis, drug delivery, and sensors. Their fluorescence can have various origins: the metal nodes, the organic linkers, or the guest molecules trapped within the pores, which can enhance or quench the fluorescence (Figure 20) [143].

Based on the detection mechanism, MOF-based sensors can be classified as (*i*) turn-on sensors, which exhibit an increase in fluorescence upon interaction with the target analyte; (*ii*) turn-off sensors, which show a decrease in fluorescence when the target analyte is present; and (*iii*) ratiometric sensors, which provide a ratio among two different fluorescence signals, offering more accurate and reliable detection.

**Figure 21:**
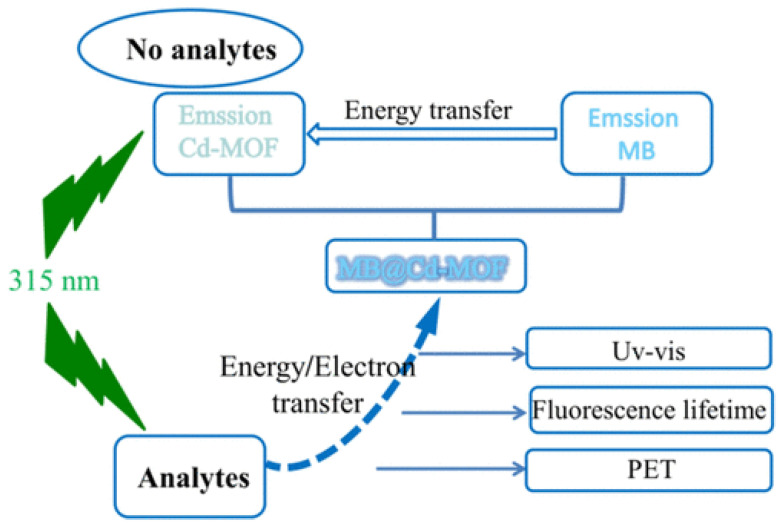
Schematic illustration of the mechanism of carbaryl sensing. Reproduced with permission from [144]. Copyright 2022, American Chemical Society.

Numerous examples of fluorescent sensors based on MOFs have been reported in the literature, particularly for applications related to environmental monitoring. In Zhang et al. [144], a stable and dual-emitting MB@Cd-MOF, highly sensitive to the pesticide carbaryl, was synthesized through a solvothermal reaction of Cd(NO_3_)_2_ salts and 3,5-di (2′,5′-dicarboxylphenyl) pyridine (H_4_DDPP), reaching an LOD of 6.7 ng·mL^−1^. The fluorescence enhancement mechanism of MB@Cd-MOF was mainly ascribed to the energy transfer from carbaryl to MB@Cd-MOF, the enhanced fluorescence lifetime after sensing carbaryl, and the PET from carbaryl to H_4_DDPP (Figure 21).

**Figure 22:**
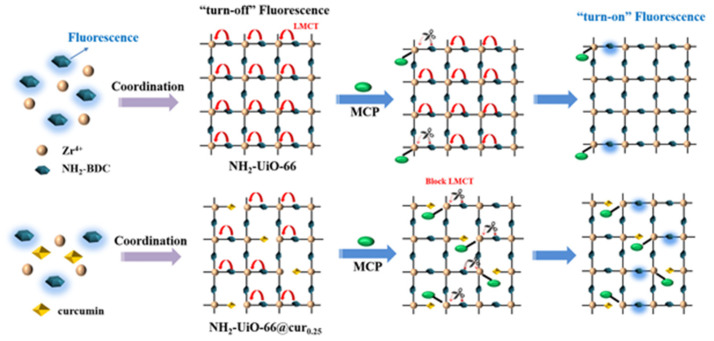
Schematic illustration of MCP detection based on “turn-on” fluorescence effect. Curcumin was used to adjust the pore size of NH-UiO-66, which promoted the mass transfer and adsorption of MCP to block the ligand-to-metal charge transfer effects, where Zr nodes served as MCP recognition sites and organic ligands as fluorescence sources [145]. Reproduced with permission from [145]. Copyright 2025, Elsevier.

Recently, Gong et al. [145] described a Zr-based MOF synthesized to detect the organophosphorous pesticide monocrotophos (MCP), in which curcumin was used to adjust the pore size of the MOF, providing more binding sites and facilitating the mass transfer and adsorption of MCP (Figure 22) [145]. The coordination between MCP and Zr nodes effectively blocked the LMCT effect, ultimately resulting in an amplified fluorescence response, with an LOD of 0.41 ng mL^−1^ (1.84 nM) in various environmental water samples.

Similarly, specific MOFs have demonstrated responsiveness to volatile organic compounds (VOCs), including formaldehyde and benzene derivatives, through guest–host interactions that alter photoluminescent signals [146]. In the context of security, MOFs functionalized with electron-deficient ligands have been employed to detect nitroaromatic explosives like TNT via fluorescence turn-off behavior. Additionally, the detection of heavy metal ions, such as Hg^2+^ or Pb^2+^, has been accomplished through ion-exchange fluorescence responses, offering promising pathways for water purification monitoring. These examples underscore the versatility of MOF-based fluorescent sensors for environmental diagnostics.

Other popular MOFs used in optical sensing over the last five years are as follows.

(1) UiO-66 is known for its stability and high surface area. UiO-66 is widely used in optical sensing applications, particularly for detecting volatile organic compounds (VOCs), heavy metals, cancer, and SARS-CoV-2 [147].
(2) ZIF-8 is known for its excellent chemical stability and large surface area. It is often used in gas sensing and detecting small organic molecules [148].
(3) MIL-101(Cr) is favored for its large pore size and high surface area, making it suitable for sensing applications involving larger molecules and pollutants [148].
(4) HKUST-1 is commonly used in optical sensing due to its unique luminescent properties and ability to detect various gases and organic compounds [149].
(5) MOF-5 is known for its high porosity and tunable structure. MOF-5 is used in optical sensors for detecting gases and environmental pollutants [149].

Despite their many advantages, there are still several challenges to be faced in MOF research, such as issues related to their stability, scalability, and functionality. Ongoing research is focused on addressing these challenges to overcome the current limitations and expand their practical uses [150].

### 2.6. Biomolecule-Based Sensors

Biomolecule-based fluorescent sensors include any fluorescent sensor incorporating biological molecules, such as proteins, enzymes, nucleic acids, antibodies, whole cells or subcellular organelles, or tissues. Coinciding with fluorescent biosensors, this group undoubtedly represents the largest one among those mentioned so far. In the present section, we therefore divide these sensors based on the biomolecules that they use for sensing.

**Figure 23:**
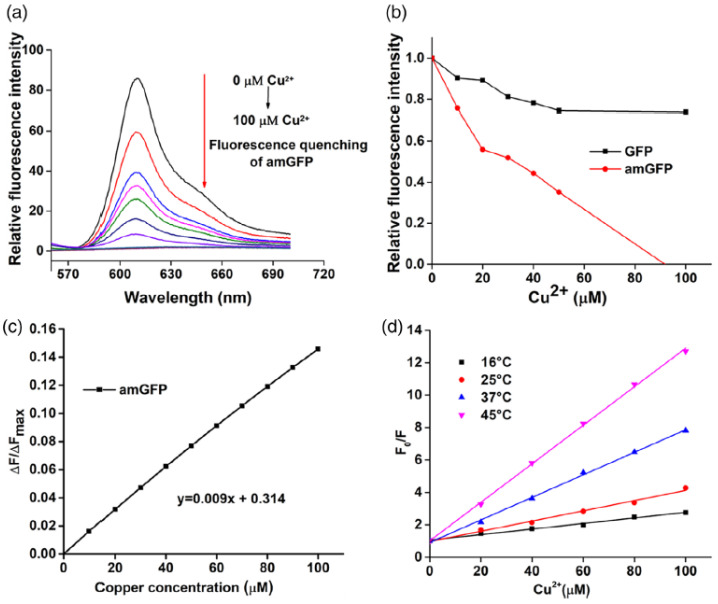
(**a**) Protein-based biosensor showing fluorescence quenching in the presence of various concentrations of Cu^2+^; (**b**) titration curves for GFP and amGFP upon addition of various concentrations of Cu^2+^; (**c**) standard curve of ΔF/ΔFmax against various Cu^2+^ concentrations for determination of Kd value; (**d**) Stern–Volmer plots of amGFP generated in the presence of copper ions at different temperatures [153]. Reproduced with permission from [153]. Copyright 2022, John Wiley and Sons.

**Figure 24:**
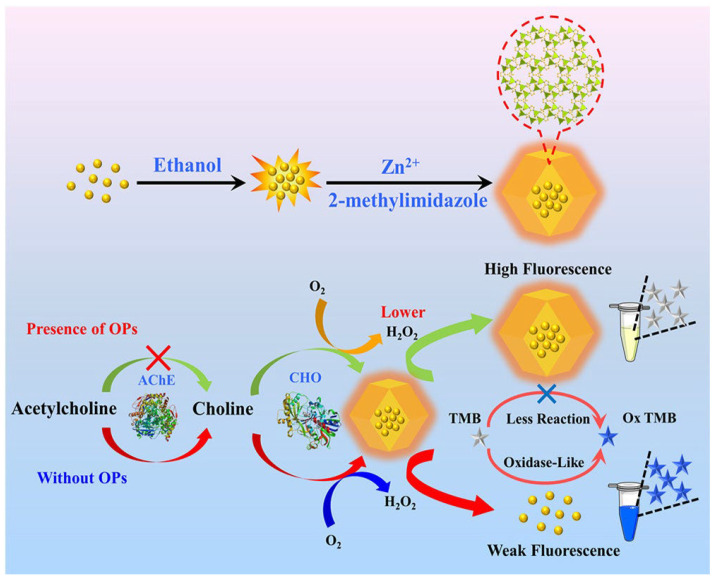
Schematic diagram of the mechanism of the detection of OPs by a AuNCs@ZIF-8-based sensor [155]. Reproduced with permission from [155]. Copyright 2021, American Chemical Society.

(1) *Protein-based fluorescent sensors*: These use fluorescent proteins such as green fluorescent protein (GFP) and its variants for the detection of ions, proteins, and nucleic acids [151] or mCherry (red fluorescent protein) and yellow fluorescent protein (YFP) to design sensors for live-cell imaging and the real-time monitoring of cellular processes [152]. In Sisila et al. [153], a Cu^2+^ sensor with high sensitivity and selectivity was developed by incorporating aminotyrosine (as an electron-donating group) into the GFP chromophore. The result was the development of an efficient red-shifted fluorescent protein-based biosensor that could also act as a bio-cleaner for the removal of Cu^2+^ ions from wastewater samples, with an estimated LOD of 1–5 μM, taking into account the calculated K_d_ of 5.25 ± 1.59 μM (see Cu^2+^ titration in Figure 23) [153].

(2) *Enzyme-based fluorescent sensors*: These use enzymes that produce a fluorescent signal upon interaction with a target biomolecule. Enzymatic fluorescent biosensors are the main tools in biosensor technology, currently used in clinical diagnosis, environmental monitoring, and industrial applications. This group of sensors includes oxidoreductases (historically used to detect glucose in the blood), transferases, hydrolases, lyases, isomerases, and ligases [154], which are associated with quantum dots, fluorescent proteins, and nanomaterial-enhanced fluorophores and allow for enhanced signal stability, multiplexing capabilities, and reduced detection limits. In Cai et al. [155], an innovative dual-mode biosensor (fluorescent and colorimetric) is described for the detection of organophosphorous pesticides (OPs). The sensor is obtained by embedding gold nanoclusters (AuNCs) inside a ZIF-8 MOF, leading to the highly fluorescent composite AuNCs@ZIF-8. In the presence of active acetylcholinesterase (AChE) and choline oxidase (CHO), acetylcholine is enzymatically converted, producing H_2_O_2_, which degrades ZIF-8 and produces a fluorescence reduction. In such a system, when OPs are present, they inhibit AChE, so less H_2_O_2_ is formed and fluorescence is retained, while the system changes color (from blue to grey), as shown in Figure 24 [155].

**Figure 25:**
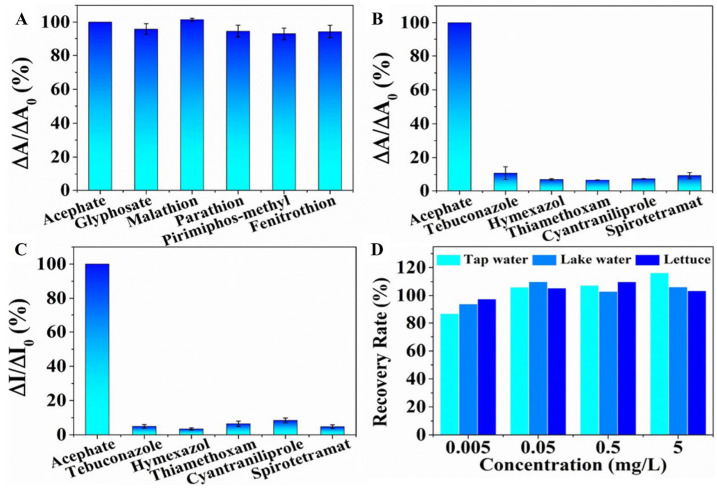
(**A**) Absorbance change at 15 mg/L in different OPs in the sensing system. Selectivity of the biosensor according to UV–vis (**B**) and fluorescence (**C**) detection. (**D**) Recovery test in water samples [155]. Reproduced with permission from [155]. Copyright 2021, American Chemical Society.

Acephate was first tested to determine the LOD, giving a value of 0.67 μg/L. To evaluate the application potential and feasibility in actual detection, the standard addition method was used for the monitoring of other OPs in water samples and lettuce extracts, all giving similar results (Figure 25) [155].

**Figure 26:**
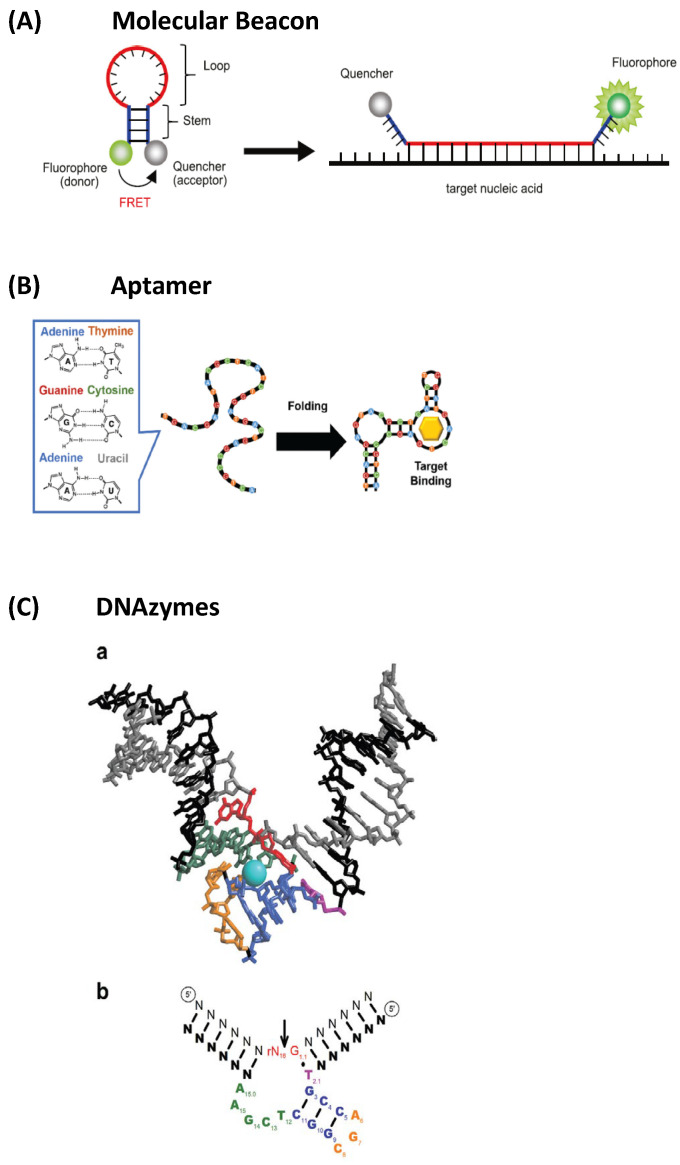
(**A**) Structure and nucleic acid detection mechanisms of MBs [156]. (**B**) Detection mechanism of aptamers—thanks to their specific base pairing, single-stranded DNA or RNA folds into unique thermodynamically favored 3D nanostructures that enable target recognition and binding [158]. (**C**) One of the best-known RNA-cleaving DNAzymes (called 8–17 motif): (**a**) overview of the three-dimensional, V-shaped structure, hosting a catalytic core (blue, orange, and green residues) around the cleavage site (located between the two nucleotides in red); (**b**) conventional representation of the secondary structure (the cleavage site is indicated by an arrow; the Pb^2+^ ion is shown as a cyan sphere) [159]. (**A**) Reproduced with permission from [156]. Copyright 2022, John Wiley and Sons. (**B**) Reproduced with permission from [158]. Copyright 1996, Springer Nature. (**C**) Reproduced with permission from [159]. Copyright 2022, The Royal Society of Chemistry.

(3) *Nucleic acid-based fluorescent sensors*: These employ fluorescent probes designed to bind specific DNA or RNA sequences, but also distinct molecules, such as proteins; thus, they can be powerful tools to elucidate intracellular processes in genetic analysis and disease diagnostics [156]. This group includes (*i*) molecular beacons (MBs) (Figure 26A, extracted from [156]), which are pioneering nucleic acid-based sensors, developed as fluorescent “turn-on” detection systems [157]; (*ii*) aptamers (Figure 26B) [158], which are oligonucleotides binding to specific target compounds or proteins; and (*iii*) DNAzymes (Figure 26C) [159], which are synthetic single-stranded DNA molecules with catalytic activity, obtained by in vitro selection, having two complementary binding arms—one able to bind to the target sequence of a substrate and the second exhibiting phosphodiester bond cleavage activity [159].

Among the various environmentally oriented applications of nucleic acid-based fluorescent sensors reported in the literature, one noteworthy example involves a paper-based fluorescence aptasensor for detecting the SARS-CoV-2 spike protein, with a detection limit as low as 0.067 ng/mL (equivalent to 0.335 pg per test) [160]. The sensing platform was engineered by immobilizing multilayer Nb_2_C MXene nanostructures, functioning as fluorescence quenchers, together with carbon dot-conjugated aptamer probes (G-CDs@Apt), onto a mixed cellulose ester (MCE) paper substrate. Within this configuration, the G-CDs@Apt probes were abundantly adsorbed onto the MXene layers, maintaining a fluorescence-suppressed state. Upon target recognition, the aptamer probes dissociated from the quencher surface, thereby restoring the fluorescence signal on the sensing paper. Additional significant cases involve proteins, metal ions, small organic molecules, and emerging environmental contaminants (such as certain viruses).

**Figure 27:**
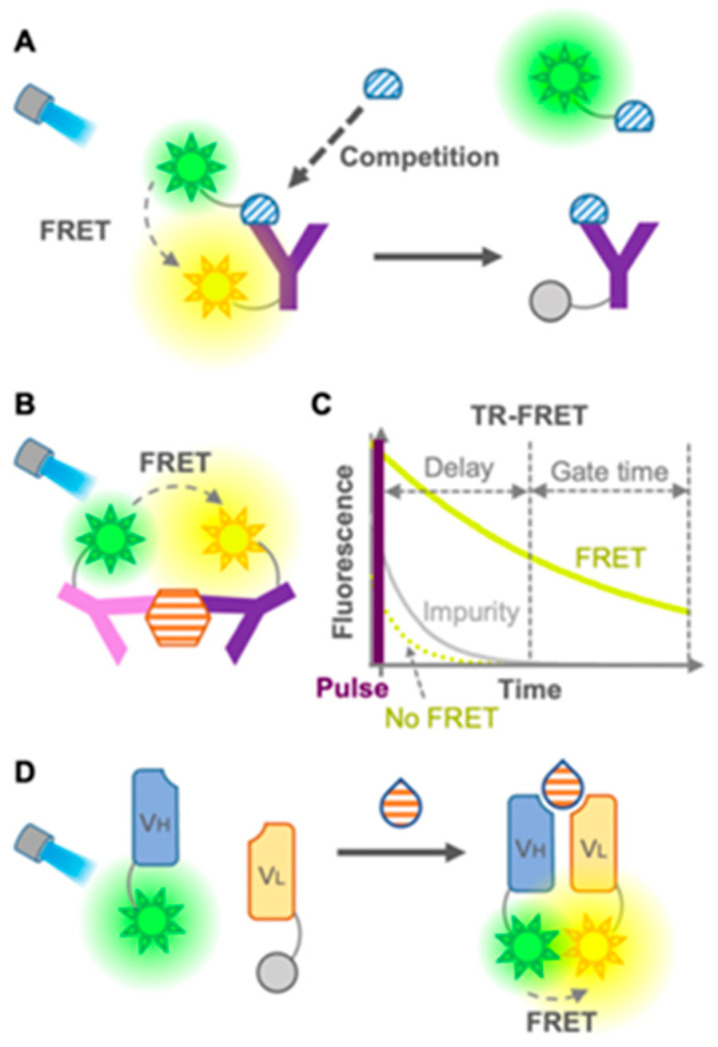
FRET immunosensors. (**A**) Scheme of a competitive FRET immunosensor. (**B**) Scheme of a non-competitive FRET immunosensor. (**C**) Principles of the TR-FRET immunoassay. Fluorescence from the acceptor (green) and impurity (gray). (**D**) Scheme of an open sandwich non-competitive immunoassay. “Y” shape: antibody; semicircle: small-molecule antigen; hexagon: large-molecule antigen; drop shape: large- or small-molecule antigen; sun shape: fluorophore [161]. Reproduced with permission from [161]. Copyright 2023, Royal Society of Chemistry.

(4) *Antibody-based fluorescent sensors*, namely *fluorescent immunosensors*: These are the most commonly and widely used immunosensors for environmental and food monitoring, as well as disease diagnosis, owing to the high sensitivity and specificity of antibodies in the recognition of their substrates [161]. Fluorescence-based immunosensors offer several key advantages, including rapid response times and non-invasiveness due to the use of light-based excitation, the ability to perform multiplexed analyses via distinct fluorescence wavelengths, and superior sensitivity and selectivity relative to colorimetric or absorbance-based methods. They can be divided into two groups: (1) FRET-based immunosensors and (2) single-fluorophore immunosensors. The first group (Figure 27) [161] includes conventional FRET sensors (donor and acceptor fluorophores on antibodies or antigens), time-resolved FRET (TR-FRET, using long-lifetime donors to reduce background noise), open-sandwich FRET (using fluorescein- and rhodamine-labeled heavy-chain (VH) and light-chain (VL) fragments for small molecule detection), and pincer-type FRET (which uses DNA duplexes to stabilize fluorophore-labeled antibody pairs).

Among FRET-based immunosensors, a recent example was reported by Pan and Wei [162], who described a FRET-based fluorescent immunosensor for the rapid and sensitive detection of dicofol, an organochlorine pesticide, using Au-Ag bimetallic nanoclusters (NCs) and gold nanoflowers (NFs). The detection platforms were developed in both liquid systems and paper-based analytical devices, and the FRET mechanism was confirmed by spectral overlap and fluorescence lifetime reduction between NCs (donors) and NFs (acceptors). Upon the binding of dicofol to the antibody-conjugated NFs, the FRET effect was disrupted, leading to fluorescence recovery proportional to the dicofol concentration. The detection limits achieved were 0.185 ng/mL in a liquid system and 0.170 ng/mL in paper-based analytical devices.

**Figure 28:**
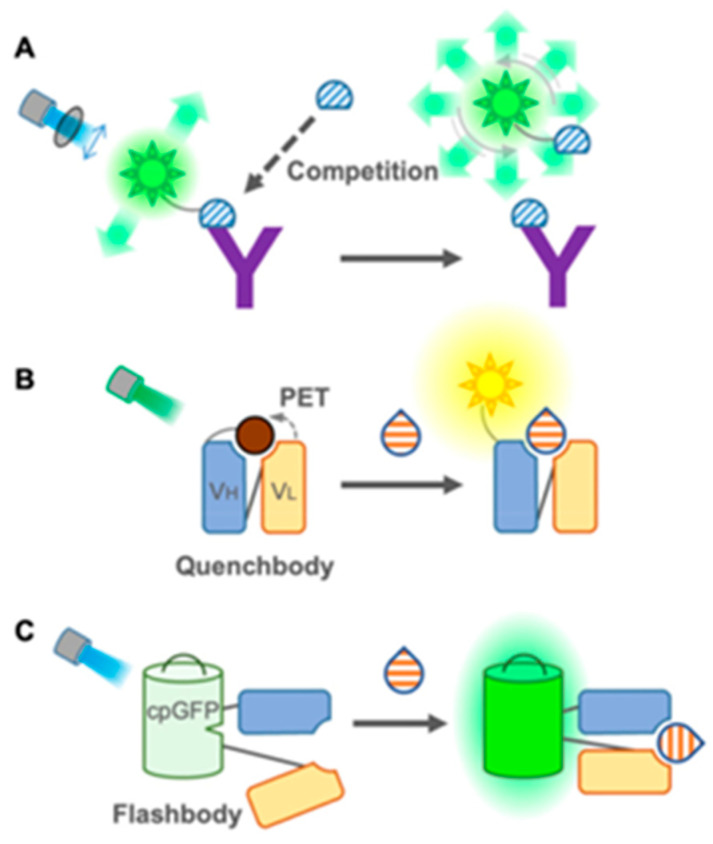
Single-fluorophore immunosensors. (**A**) Scheme of a polarization-based immunosensor. (**B**) Scheme of a quench-body (Q-body) immunosensor. (**C**) Scheme of a flashbody immunosensor. “Y” shape: antibody; semicircle: small-molecule antigen; drop shape: large- or small-molecule antigen; sun shape: fluorophore [161]. Reproduced with permission from [161]. Copyright 2023, Royal Society of Chemistry.

The group of single-fluorophore immunosensors (Figure 28, from [161]), which use only one fluorophore and rely on changes in intensity or polarization, includes the fluorescence polarization immunoassay (FPIA—detecting binding-induced changes in molecular rotation), quench body (Q-body—fluorophore quenched by tryptophan residues and unquenched upon antigen binding), coiled Q-body (a modular version using peptide pairs for flexible dye labeling), and flashbody (a genetically encoded sensor using circularly permuted GFP inserted into antibody fragments).

An interesting FPIA has been recently realized by Zhou et al. [163] for the detection of imidacloprid (a neonicotinoid insecticide) in environmental samples. The work describes six fluorescent tracers synthesized by conjugating haptens of imidacloprid, acetamiprid, and thiamethoxam with fluorescein derivatives—either ethylenediamine fluorescein (EDF) or aminomethyl fluorescein (AMF). Among them, the IMI-EDF conjugate exhibited the greatest increase in fluorescence polarization upon binding to the anti-IMI monoclonal antibody and was therefore selected as the optimal tracer for assay development, showing an LOD of 1.7 µg/L.

(5) *Fluorescent sensors based on living (micro)organisms or parts of them*: This group of biosensors includes any living (micro)organism, or part of one (single cell, organelle, tissue), with unaltered vitality, naturally able to emit fluorescence or genetically engineered to produce fluorescent signals in response to specific stimuli. There are a number of articles in the literature reporting sensors of this type. Due to their large number, we only report here the latest trends for each mentioned subtype, considering (*i*) whole-cell biosensors, (*ii*) organelle-based biosensors, and (*iii*) tissue-based biosensors.

Whole-cell biosensors use both prokaryotic and eukaryotic organisms to detect environmental and biomedical targets. These biosensors can be broadly categorized into two groups: natural whole-cell biosensors and genetically engineered whole-cell biosensors.

Natural whole-cell biosensors involve the interaction of the target directly with cell metabolites, cell membrane molecules, or cell walls. Six families of cells are described in the literature as sensing elements for this type of biosensor [164]. The main one is the bacterial family, used since 1965 [165]. This is followed by fungal/yeast cells [166] and microalgae [167], as well as animal [168] and plant cells [169]. Over the past 10 years, bacterial viruses (bacteriophages) have emerged [170], which are used for the detection of pathogenic bacteria in healthcare, food, and the environment. The choice of one family of cells over another will depend not only on the detection capabilities, the robustness of the cell during use, and its storage capacity but also on the ease of its production.

**Figure 29:**
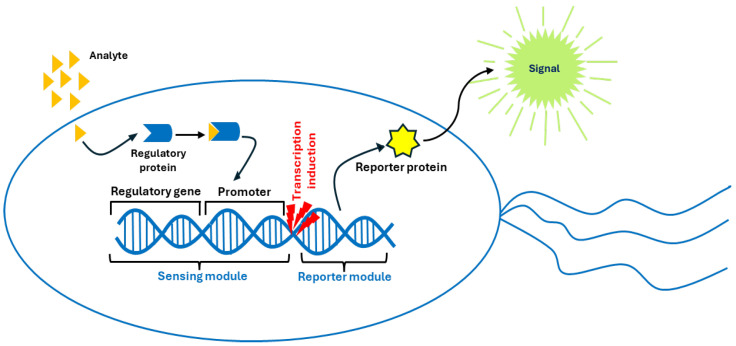
Schematic representation of a genetically engineered prokaryotic cell biosensor. Two main components can be distinguished within the cell: the sensing module (composed of a regulatory gene fused to a “promoter”, i.e., a fragment induced by the target analyte through a regulatory protein) and the reporter module (which consists of genes coding for optically active proteins, such as GFP, RFP, or LuxCDABE, which are expressed only when the target analyte is present, generating a fluorescence output signal).

Genetically engineered whole-cell biosensors have emerged as a low-cost, high-specificity, high-sensitivity, and rapid alternative to biological receptors [171]. They essentially involve the expression of certain crucial genes regulated and promoted by other segments of the cell genome. This method employs the tools of synthetic biology for sensor development and regulates the expression of genes to monitor analyte concentrations. In general, a genetically engineered prokaryotic cell biosensor (see Figure 29) has two important components: the sensing module (responsible for the identification of target analytes and regulation of transcription) and the reporter module (responsible for generating an output signal). Sensing and reporter modules can be mediated with genetic circuits (which can be composed of toggle switches, logical gates, or amplification circuits) to improve the various parameters of a biosensor [172].

Similarly, genetically modified eukaryotic cell-based biosensing systems generally involve specific receptors activated by target analytes, which are coupled with a reporter gene, inducing an output signal.

**Figure 30:**
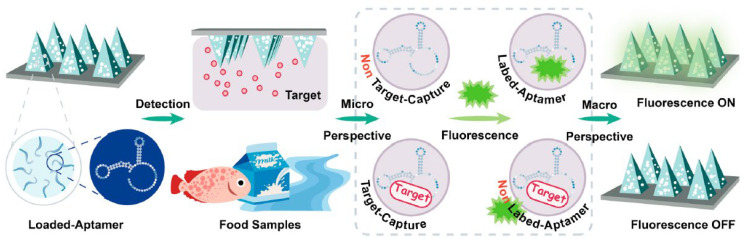
Schematic illustration of PMN with fluorescent aptasensor for antibiotic residue detection in food [176]. Reproduced with permission from [176]. Copyright 2024, Elsevier.

Organelle-based biosensors. These biosensors leverage the unique properties of organelles to detect specific biochemical changes or monitor cellular processes, ultimately providing higher selectivity because of their direct target analyte recognition. Among the organelles used as bioreceptors, there are, for example, mitochondria, which have been immobilized on optical transducers to monitor, through their membranes, reactive oxygen species (ROS) production, oxidative stress, and metabolic activity [173]. Another example of organelles, exploited in the biosensor area since the 1990s, is represented by thylakoids and chloroplasts, which are responsible for photosynthesis in plants and can be utilized to monitor environmental pollutants, such as herbicides [174]. In 1996, Merz et al. [175] developed a biosensor based on isolated chloroplasts from higher plants, able to detect triazine and phenylurea herbicides in drinking water with a limit of detection of 0.1 μg/L per single herbicide, as required by European Community legislation on drinking water quality. In a recent work employing a distinctly modern methodological framework [176], photosynthetic oxygen evolution from spinach’s thylakoid membranes was used to realize a sustainable, bioinspired method of fabricating porous microneedles (PMNs) (Figure 30) [177]. These PMNs are functionalized with a fluorescent aptasensor targeting netilmicin (NET), an aminoglycoside antibiotic. Upon NET binding, the fluorescence decreases due to aptamer structural changes, providing a quantifiable signal, with high sensitivity (LOD: 5.99 nM).

Tissue-based biosensors. Tissue-based biosensors have several advantages in various fields, such as in the treatment of diseases, drug delivery, tridimensional printing, tissue engineering, and genetic therapy. In general, they can be formed from genetically modified cells or by direct genetic modification to introduce biosensor proteins (able to interact with the target of interest) into a tissue in the animal. Biophotonics provides the most versatile basis for tissue-based biosensors. Light output from biosensor cells can be in the form of fluorescence or bioluminescence, and, of these two, bioluminescence offers the advantages of not requiring an input source of light and having a more favorable signal-to-noise ratio in living animals than fluorescence. Protein–protein interactions can be used to detect almost any molecule by means of fusion proteins that can be used to generate resonance energy transfer. Bioluminescence resonance energy transfer (BRET) has the potential to be used for the measurement of a wide variety of molecules in living animals. In Acha et al. [178], a very interesting tissue-based biosensor for the hormone vasopressin is discussed, in which the use of a plastic window in the skin/body walls of mice permits measurements of light produced by bioluminescent cells transplanted into the kidney. The cells coexpressed firefly luciferase FLuc, a vasopressin receptor–Renilla luciferase RLuc fusion protein, and a GFP2–arrestin2 fusion protein. Following the intraperitoneal injection of vasopressin, a marked increase in BRET was measured via the window, using fiber optics and a photon counter.

In another experiment carried out in the field of tissue-based FRET biosensors, Bouchala et al. [179] designed a biosensor in which nanometric fluorescent nanoemulsion droplets encapsulating lipophilic near-infrared cyanine dyes of phenylborate were used for the in vivo imaging of mice. In this experiment, they found that FRET nanocrystals in blood circulation were preserved at 93% for 6 h, but this dropped to 66% in the liver, showing that tissue-based FRET biosensors can provide important information about the distribution, activity, and expression of biomolecules in tissues, helping to understand biological processes and identify new targets for therapeutic intervention. These achievements, while appearing strictly related to the biomedical field, offer powerful insights that can be translated into environmental biosensing for both the detection and dynamic tracking of environmental pollutants, with direct observations of the effects produced on living organisms. However, the potential of tissue-based biosensors, notably optical ones, remains largely underexploited, except for a few examples, such as liver-on-a-chip systems (first realized to detect drug toxicity), which have been adapted to monitor waterborne toxins like aflatoxins.

In summary, organic fluorescent sensors have become vital analytical tools in the field of environmental monitoring, offering the rapid, sensitive, and non-destructive detection of a wide array of pollutants, including heavy metals, pesticides, VOCs, and endocrine-disrupting chemicals. Their molecular tunability enables the precise recognition of target analytes, while their compatibility with portable and low-cost platforms facilitates real-time, on-site analysis in complex environmental matrices. Advances in molecular design, such as photoinduced electron transfer (PET) and intramolecular charge transfer (ICT) mechanisms, have significantly improved the detection limits and selectivity, even in the presence of potential interferents. Despite challenges related to photostability, matrix interference, and long-term field deployment, innovations in synthetic fluorophore design, nanomaterial integration, and AI-assisted signal interpretation are poised to further strengthen the functionality, sustainability, and intelligence of these systems, paving the way for smarter, greener, and more accessible sensing technologies. As such, organic fluorescent sensors represent a promising, sustainable alternative to conventional instrumentation for decentralized, high-resolution environmental diagnostics.

Table 1 summarizes the most recent applications of fluorescent sensors based on organic materials and the related LODs.

## 3. Insights into Inorganic Alternatives

**Figure 31:**
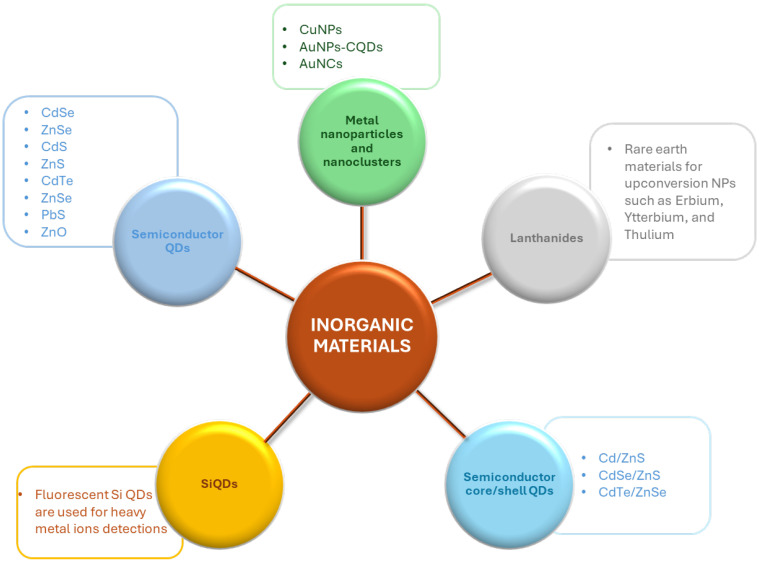
Inorganic materials used as fluorescent sensors.

Inorganic nanoparticles have drawn considerable attention as fluorescent sensors in different applications, such as environmental analysis. Among them, some of the most widely studied are semiconductor quantum dots (QDs) of the III-V, II-VI, and IV-VI periodic groups (e.g., CdSe, ZnSe, CdS, ZnS, CdTe, ZnSe, PbS, etc.), metal nanoparticles, lanthanides and other transition metal nanoparticles (mostly as upconversion fluorescent sensors), and Si QDs, as summarized in Figure 31.

Nanomaterial synthesis is traditionally categorized into bottom-up and top-down approaches. However, several recent methods operate at the intersection of these classifications. Bottom-up approaches involve assembling atoms and/or molecules into larger structures, similar to a Lego-like process. In contrast, top-down methods rely on removing material from a bulk source to create the desired nanostructure, resembling a sculpture-like technique [203].

QDs offer high flexibility in terms of the emitting wavelength thanks to the quantum confinement effect, enabling the engineering of the fluorescence wavelength according to the size of the particles. Other advantages of quantum dots are typical narrow light emission, a wider range of absorbance, and a larger Stokes shift compared to traditional dyes [204,205]. Moreover, core–shell QDs show improved quantum yields and stability under photobleaching, reducing electron–hole pair recombination [206,207].

Semiconductor QDs such as CdSe, CdTe, and PbS are the most investigated inorganic fluorescent sensors in the literature thanks to their high quantum yields and stability, which have enabled these structures to be used successfully for several applications [208]. However, a well-known drawback of these nanostructures is their toxicity due to the heavy metal ions contained. Indeed, CdSe, CdTe, PbS, and so on have also been investigated as secondary pollution causes. Novel strategies such as bioconjugation approaches have been proposed to overcome these issues, improving their application as analytical sensors [209]. In the last few years, core–shell structures such as Cd/ZnS have been some of the most investigated ones thanks to their high engineerability in terms of fluorescence wavelengths, quantum yields, and lifetime. Another crucial reason for their success lies in the efficient passivation of the surface trap state given by the shell, leading to high and robust quantum yields [210,211].

CdSe/ZnS quantum dots are largely used as photoluminescence (PL) sensors for pH determination, and several works report their pH sensing in the 3–10 pH range when using different coatings [212,213,214,215,216]. The sensing mechanism relies on PL turn-on, PL quenching, FRET, and ratio fluorescence. For example, Li et al. used a p-aminothiophenol coating for CdSe/ZnS core–shell quantum dots and demonstrated turn-on PL pH detection in aqueous media, obtaining a linear response in the 3.2–6 pH range. In this work, the authors showed an application with filtered tap water, validating their results by using a pH meter [217], demonstrating that similar sensors may be applied for real purposes.

**Figure 32:**
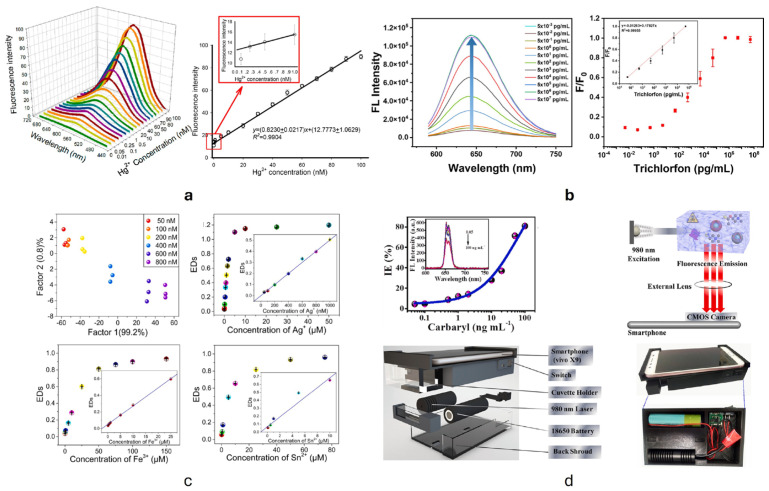
(**a**) Fluorescence intensity with respect to Hg^2+^ ion concentration and fluorescence calibration curve from 0.1 to 100 nM. In the inset, a detail of the calibration curve from 0.1 to 1 nM is shown [224]. Reproduced with permission from [224]. Copyright 2020, American Chemistry Society. (**b**) Emission spectra of a DNA-AgNC-based sensor at different pesticide concentrations and the fluorescence trend as a function of the pesticide concentration. In the inset, the concentration range is shown, where a linear response from the sensor is observed [225]. Reproduced with permission from [225]. Copyright 2025, American Chemistry Society. (**c**) A canonical score plot for the fluorescence response vs. different concentrations of Ag^+^ ions, as well as ED values vs. different concentration ranges of cation analytes [234]. Reproduced with permission from [234]. Copyright 2021, Elsevier. (**d**) Fluorescence intensity vs. concentration of carbaryl, from 0.05 ng/mL to 200 ng/mL, and the portable experimental setup, consisting of a 980 nm laser for excitation, a cuvette holder, and a smartphone as a detector [235]. Reproduced with permission from [235]. Copyright 2021, Elsevier.

Semiconductor QDs are also used for heavy metal trace detection. For example, Marukhyan et al. reported the use of urease in combination with CdSe as an enzymatic sensor for the detection of several metal traces in water, such as Co, Mg, Fe, Cr, Zn, and Ni. The sensor is based on the inhibitory effects of these metals on urease activity, which therefore influences the pH, causing a fluorescence variation in CdSe quantum dots [218]. Farahmandzadeh et al. reported the application of CdTe/ZnSe core–shell quantum dots as turn-off fluorescent sensors for the detection of lead ions in an aqueous medium. For these CdTe/ZnSe quantum dots, thioglycolic acid (TGA) was used as a capping agent. TGA has a thiol functional group in its structure, and, due to the strong affinity of lead ions for thiol groups, they effectively compete with the ZnSe shells of the quantum dots (QDs) and form robust covalent bonds with multiple TGA molecules. This interaction results in the detachment of the TGA molecules from the nanoparticle surface and the aggregation of the CdTe/ZnSe QDs, causing the PL quenching mechanism. This effect is also responsible for the selectivity of these CdTe/ZnSe QDs, which have negligible PL quenching with other heavy metals and ions, such as Mo^4+^, Na^+^, S^2−^, Zn^2+^, Cd^2+^, Sr^2+^, Ca^2+^, Mn^2+^, Fe^3+^, and so on. The authors found an LOD of 31.8 nM, with a linear response trend in the 50 nM–10 µM range, and conducted successful tests in real aqueous media, including tap water, river water, and agricultural water, demonstrating the use of these QDs also immobilized in glass slides. Several other authors [219,220,221,222] have demonstrated similar performance for Pb^2+^ with other fluorescent sensors based on QDs [223]. Yi Zhang et al. developed a highly selective sensor for Hg^2+^ detection using biosynthetic CdSe/CdS QDs combined with a liposome carrier as a fluorescence amplifier. The sensor demonstrated a detection limit of 0.01 nM and an effective detection range from 0.25 to 100 nM [224] (Figure 32a).

Commercial CdSe/ZnS quantum dots have also been used in combination with DNA-templated Ag nanoclusters as a colorimetric and fluorescence pesticide sensing platform (Figure 32b). A linear trend was shown, as the photoluminescence increased as a function of the trichlorfon concentration in the 5–5 × 10^5^ pg/mL range, with an LOD of 2.55 pg/mL for the DNA-templated Ag nanocrystal samples [225]. When CdSe/ZnS is combined with these sensors, a colorimetric change through an ion exchange process between QDs and DNA-AgNCs leads to a colorimetric response, with a linear trend in the 0.05–5 × 10^10^ pg/mL range and a 1.06 pg/mL LoD. The same sensors were also tested with different interferent species, demonstrating excellent selectivity, and were finally used in the detection of trichlorfon in tap and local river water samples, without any particular issue [225].

III–V and II–VI QDs are some of the most widely described fluorescent sensors in the literature, and their superior quantum yields as well as robustness have encouraged research. Nonetheless, other different strategies have been investigated, and the application of other materials for QDs or nanostructures is also reported in the literature. We can find studies reporting ZnO quantum dots [226], metal nanoclusters [207], upconverting nanoparticles based on rare earth materials [227], and also fluorescent perovskite quantum dots [228].

ZnO quantum dots are also used as fluorescent sensors, offering an alternative to the more common III–V or II–VI semiconductor QDs. Their appeal lies in their good biocompatibility, low material costs, and environmentally friendly degradation products, which show no toxic effects, while maintaining good performance in fluorescent sensing, even with lower quantum yields compared to the previously discussed QDs [226].

Metal nanoclusters (NCs) are nanoparticles typically smaller than 2 nm, showing discrete densities of energy due to quantum confinement effects [229]. Different from larger metal nanoparticles, which are often characterized by plasmonic effects when their size is reduced to less than 2 nm, unique chemical and physical properties arise [230,231,232], such as intense photoluminescence, often described in terms of HOMO and LUMO molecular-like behavior, which has also been used for sensing applications. For example, gold nanoclusters—hybrid systems based on gold nanoparticles or gold nanoclusters—have promising applications as fluorescent sensors for detecting contaminants in food [233] and heavy metal ions (Figure 32c) [234]; a similar mechanism to the one described above has been employed for pesticide detection in a portable device, using a smartphone as the detector (Figure 32d) [235]. Fluorescent CuNPs are also well known for heavy metal detection, such as Hg^2+^, Pb^2+^, Cu^2+^, and Fe^3+^ [207,236].

Rare earth metals such as erbium, ytterbium, and thulium are often used as dopants in the crystal lattices of other dielectric lanthanides, or transition metals such as scandium and yttrium can be used to obtain fluorescent upconverting NPs (Figure 32d). Lanthanide ions are typically found in their trivalent state and are characterized by narrow and well-structured emission bands. Other advantages are a long photoluminescence lifetime, a large Stokes shift, and exhibiting photon upconversion [227,237,238,239,240]. Upconversion is a non-linear optical process consisting of the capability of these ions of absorbing multiple photons in the near-infrared range, re-emitting light in the UV–visible range [241,242]. Indeed, rare earths in their normal states are characterized by weak emission due to the forbidden f-f transitions [240,243]. These transitions become allowed when they are ionized inside proper crystal lattices through doping, and this is the same strategy used for erbium ions inside fiber optics for long-range telecommunications. The crystal lattice used as a host is chosen as a transparent material that allows a lower mismatch with the original emitter lattice, avoiding efficiency losses due to non-radiative recombination [244]. Lanthanide dopants are commonly employed in pairs, using one as the activator (e.g., Er) and the other as the sensitizer (e.g., Yb) [245]. The emission wavelength depends on the chosen doping lanthanide, enabling different available wavelength emissions [242,246].

Silicon is an indirect-bandgap material, and its light emission at room temperature is negligible, making it an inefficient light-emitting material [247,248,249,250,251]. Obtaining light emission from Si-based materials has been a central topic in photonics for several years, and different strategies involving impurity doping and quantum confinement effects have been investigated by the scientific community [250]. Apart from impurity doping [252] or defect emission [253], some of the first works reporting significant light emission presented the fabrication of Si nanocrystals embedded in a silica matrix [254] by using top-down approaches. In these works, the photoluminescence of Si was obtained by quantum confinement effects, demonstrating the dependence of the emission wavelength on the size of the QDs [255]. However, this type of structure is not ideal for sensing purposes, complicating the interaction of the target with the fluorescent Si QDs. More recent studies have demonstrated the synthesis of Si QDs by wet self-assembly approaches, with large-scale capabilities and interesting light emission at room temperature [256]. Several works in the literature report the use of fluorescent Si QDs for heavy metal ion detection [256]. For example, Li et al. [256] demonstrated a fluorescent sensor for Hg^2+^ ion detection based on Rox-labeled DNA-functionalized Si QDs, obtaining an LOD of 0.01 nM with a linear response in the 0.02 nM–10 nM range. These performance outcomes are similar to what can be obtained with other nanostructure-based fluorescent sensors, as shown in Table 2.

Regarding silicon, Si NWs have also been studied in recent years for various fluorescent sensor applications [247], serving as substrates, quenchers, and active fluorescent transducers [250]. Some studies report their use in detecting heavy metal traces, highlighting their potential despite their primary focus on biomedical applications. However, their use as fluorescent sensors remains relatively limited, with most applications targeting biomolecules and a few studies exploring gas sensing [242].

Among inorganic materials, an emerging and particularly promising approach in recent years has been the use of 2D platforms [257], such as graphene, MoS_2_, and MXenes, which have demonstrated remarkable performance in terms of fluorescence quenching efficiency, signal amplification, and overall sensing sensitivity [258,259].

It is possible to conclude that a detection range within nM–uM and sub-nM LOD capabilities can be easily obtained for heavy metal trace detection in aqueous media by using inorganic fluorescent sensors, with several sensors already being tested in real samples. However, it should be highlighted that, in general, inorganic alternatives may outperform organic sensors in terms of performance, but organic-based sensors remain highly attractive due to their low cost, ease of functionalization, and versatility across different sensing environments. In many practical contexts, the optimal choice depends on the specific sensing application, which determines the balance between the fabrication cost, the reliability and stability of the platform, and the requirements of the target analyte.

**Table 2:** Inorganic fluorescent sensors.

Inorganic Material	Target	Technique	Type of Sample	Performance	Ref.
CdSe/ZnS QDs	pH	PL	Aqueous media	3.2–6 (pH scale)	[217]
CdSe QDs	Cu, Co, Mg, Fe, Cr, Zn, Ni ions	PL restoring	Aqueous media	LOD 0.2 µM	[218]
CdTe/ZnSe QDs	Pb^2+^	PL quenching	Real sample	LOD 31.8 nM 50 nM–10 µM (detection range)	[223]
CdSe/CdS QDs	Hg^2+^	PL restoring	Real sample	LOD 0.01 nM 0.25–100 nM (detection range)	[224]
CdSe/ZnS QDs	Trichlorfon	PL	Real sample	LOD 2.55 pg/mL 5–5 × 10^5^ pg/mL (detection range)	[225]
		Colorimetric analysis		LOD 1.06 pg/mL 0.05–5 × 10^10^ pg/mL (detection range)	
NaErF_4_-0.5% Tm^3+^/NaYF_4_	Carbaryl	PL upconversion	Extracted sample	LOD 0.05 ng/mL 0.05–100 ng/mL	[235]
CuNPs	Hg^2+^	PL restoring	Real sample	LOD 0.1 nM 0.5–100 nM (detection range)	[260]
	Pb^2+^	PL quenching	Real sample	LOD 5 nM 5–100 nM (detection range)	[261]
	Cu^2+^	PL restoring	Real sample	LOD 0.3 ppm 0.95–6.35 ppm (detection range)	[262]
	Fe^3+^	PL quenching	Real sample	LOD 10 nM 10 nM 10 μM (detection range)	[263]
	Cr(VI)	PL quenching	Aqueous media	LOD 65 nM 0.2–60 μM (detection range)	[264]
Si QDs	Mg^2+^	PL two-band ratio	Aqueous media	LOD 0.01 nM 0.02–10 nM (detection range)	[256]
Si QDs	Co^2+^	PL quenching	Real sample	LOD 0.37 µM 1–120 µM (detection range)	[265]
S-Si QDs	Fe^3+^	PL quenching	Real sample	LOD 0.21 μM 1–20 μM (detection range)	[266]
NaYF_4_ UCNPs *	Temperature	PL upconversion	Aqueous media	20–45 °C	[267]
NaBiF_4_-Yb, Er NPs	Temperature	PL two-band ratio	Aqueous media	303–523 K (detection range)	[268]

* Upconversion nanoparticles.

From a performance perspective, this technology appears well developed, offering various alternatives in terms of materials, functionalization procedures, fluorescent sensing mechanisms, and analytical strategies. However, the absence of a cost-effective nanomaterial-based assay in the commercial market suggests that further advancements in material synthesis and assay fabrication are needed to achieve commercial success, and these points will be analyzed in the next section.

## 4. Industrial Applications of Fluorescence-Based Optical Sensors

A crucial aspect of industrial applications utilizing optical sensors has been advancements in fluorescence-based optical biosensor technologies. These developments have enabled continuous and rapid progress, primarily due to the non-contact nature of these sensors, which enhances the robustness and reliability of the applications.

Among the main key technologies, there are fiber optic fluorescence sensors, which are widely used for the real-time detection of biochemical markers in both industrial and environmental monitoring. In industrial settings, these sensors are used to monitor the presence of hazardous chemicals in manufacturing plants, ensuring worker safety and regulatory compliance, while, in environmental monitoring, they are employed to detect pollutants in water bodies, such as heavy metals or organic contaminants, providing crucial data for environmental protection efforts [269,270]. Another technology is represented by fluorescence immunoassays, which are essential in pharmaceutical quality control and medical diagnostics. They are used to detect a wide range of biomarkers, pathogens, and toxins [208]. For example, in medical diagnostics, they can identify the presence of specific proteins associated with diseases like cancer or infectious agents like bacteria and viruses. In pharmaceutical quality control, they ensure the purity and potency of drugs by detecting any contaminants or degradation products [271]. Quantum dot-based fluorescence sensors represent another fully exploited technology: as stated before, these sensors offer enhanced sensitivity and are used in food safety and biomedical applications. In food safety, quantum dot-based sensors can detect contaminants such as pesticides, heavy metals, and pathogens in food products, ensuring that they are safe for consumption. In biomedical applications, they are used for imaging and in detecting specific biomolecules within cells, aiding in disease diagnosis and research [272,273]. Fluorescence resonance energy transfer (FRET) sensors are another example of industrially used sensors. They are highly valuable in biopharmaceutical applications as an alternative to standard approaches to monitoring molecular interactions, with high specificity [274]. They are used to study protein–protein interactions, enzyme activity, and other molecular processes. For example, FRET sensors can be used to observe the binding of a drug to its target protein, providing insights into the drug’s mechanism of action and efficacy [275].

**Figure 33:**
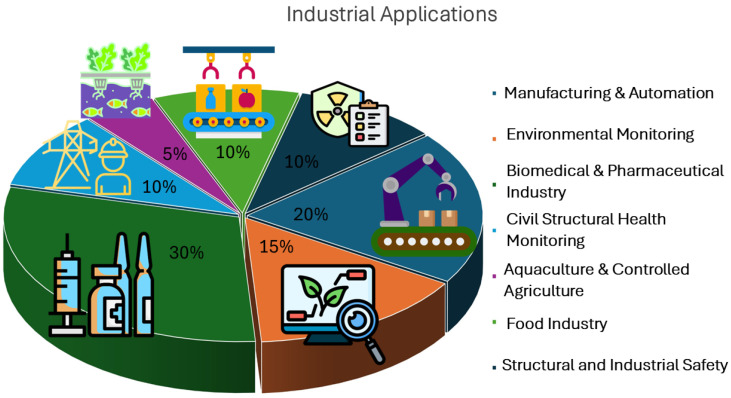
Market shares related to fluorescent-based optical sensors for industrial applications.

From an industrial perspective, the applications of optical fluorescence sensors are diverse, spanning from the food industry to environmental monitoring. Environmental monitoring, particularly in marine settings, presents greater complexity. Figure 33 illustrates the various applications and their respective market share percentages for optical fluorescence sensors. Additionally, each type of industrial application is detailed comprehensively.

Optical sensors play a crucial role in manufacturing and automation, as well as process control and robotics. Machine vision systems, integrated with optical sensors, enable defect detection, dimensional measurement, and real-time quality assessment in industries like automotives and electronics [276]. They account for approximately 20–25% of the optical sensor market in this sector [277].

In environmental monitoring, spectroscopic and fiber optic sensors are widely used to detect pollutants, monitor air and water quality, and assess industrial emissions. Fluorescence-based sensors offer additional advantages in detecting contaminants such as heavy metals, pesticides, and organic compounds in water samples, providing real-time data for regulatory compliance and environmental protection [278]. They represent about 15–20% of the market share in environmental monitoring applications [279]

Optical sensors are also widely used in biophotonics, particularly in the biomedical and pharmaceutical industries, facilitating non-invasive diagnostics, precise sensing, and pharmaceutical quality control. Fluorescence spectroscopy is particularly effective in detecting biomolecules and drug composition analysis. These sensors can identify bacteria, toxins, and allergens in medical diagnostics and food safety applications [208,280]. They hold a significant share, around 30–35%, in the biomedical and pharmaceutical industry [279].

Fiber Bragg grating (FBG) sensors are extensively used in civil structural health monitoring for bridge, pipeline, and aircraft structure supervision. These sensors provide real-time data on strain, temperature, and vibrations, ensuring early fault detection and predictive maintenance [281]. They account for approximately 10–15% of the market [279].

Fluorescence-based sensors have shown potentialities in aquaculture and controlled agriculture applications, monitoring water quality in open-sea aquaculture and recirculating aquaculture systems (RAS) [282], detecting bacterial contamination, and ensuring environmental sustainability. These sensors can also be used in controlled agriculture, where they are used to assess plant health by measuring the fluorescence emitted by chlorophyll, providing the early detection of plant stress factors [283]. They represent about 5–10% of the market in aquaculture and controlled agriculture.

Fluorescence-based sensors are also widely used in food industry applications, such as food quality and safety control assurance. They are applied in beverage differentiation (e.g., alcoholic beverages) by detecting compounds such as tannins, polyphenols, and resveratrol. Additionally, these sensors can monitor the aging processes of spirits stored in different materials [284]. They account for approximately 10–15% of the market share in the food industry [277].

Fluorescence-based optical sensors are increasingly used in structural and industrial safety for the monitoring of structural integrity and ensuring industrial safety. They represent about 10–15% of the market in this sector. These sensors and biosensors continue to evolve, being integrated with AI and nanotechnology to enhance the accuracy, efficiency, and applicability across industries [285].

## 5. Advantages, Challenges, and Perspectives of Fluorescence-Based Optical Biosensors

Fluorescence-based optical sensors offer distinct advantages—notably their high sensitivity and specificity—which enable the detection of trace analytes with minimal interference. A further strength lies in their real-time and non-invasive operation, allowing for continuous monitoring without disrupting the process flow. Although the versatility and miniaturization of these sensors has truly broadened their application landscape, making them adaptable across various industrial sectors, from healthcare to environmental safety, the full potential of these sensors emerges through their integration with artificial intelligence and machine learning, which significantly enhances data interpretation, pattern recognition, and predictive monitoring capabilities [285]. Despite their advantages, fluorescence-based optical biosensors face several challenges. These include issues related to sensor stability under industrial conditions, interference from background fluorescence, and the need for advanced calibration techniques. Ongoing research and technological advancements are addressing these challenges, paving the way for more robust and reliable biosensors. The future perspectives for these sensors are therefore promising, with potential applications expanding into new areas such as personalized medicine, smart agriculture, and advanced environmental monitoring systems. Future research will focus on enhancing sensor robustness, developing multiplex detection capabilities, and integration with smart technologies like artificial intelligence and photonics [286]. The future of fluorescence-based optical sensors in industrial applications looks promising, driven by continuous advancements in technology and increasing demands across various sectors. Key trends include miniaturization and integration with Internet of Things (IoT), with smaller, more compact sensors, integrable into IoT devices for enhanced functionalities in smart homes, industrial systems, and wearable devices [287]. Advancements in sensor technology, with new developments in fiber optic and photonic sensors, are improving the sensitivity and performance, making them ideal for harsh environments and precise measurement in fields like structural health monitoring and environmental sensing [288]. Optical sensor use is also expanding in healthcare and biomedical applications, and fluorescent sensors are becoming crucial in healthcare for non-invasive diagnostics, biomedical imaging, and remote patient monitoring, contributing to early disease detection and better patient outcomes [286]. Integration with Industry 5.0, and the adoption of optical sensors in Industry 4.0 and the Industrial Internet of Things (IIoT), is transforming manufacturing and automation by enabling real-time monitoring, predictive maintenance, and enhanced operational efficiency [288]. This trend has become very important, especially due to the number of investments made regarding the growing demand in the defense and aerospace sectors, which are increasingly relying on optical sensors for applications such as missile guidance, aircraft structural monitoring, and navigation systems, due to their robustness and precision [288]. Overall, the ongoing research and development in fluorescence-based optical sensor technologies is expected to overcome the current challenges, such as environmental susceptibility and high costs, paving the way for more robust and cost-effective solutions [287].

## 6. Conclusions

Organic fluorescent sensors have emerged as pivotal tools in environmental analysis, due to their tunable structures, high sensitivity, and variety of functionalization strategies. This review, through the many examples of organic sensors provided, has illuminated both their strengths and persistent limitations, such as photobleaching, narrow excitation ranges, and vulnerability to environmental interference, highlighting the need for continuous refinement in molecular design and probe stability. The inorganic alternatives discussed here, such as quantum dots and lanthanide-doped upconversion nanoparticles, have demonstrated remarkable optical performance and greater structural stability and environmental resilience compared to several organic sensors. Their robustness, extended emission lifetimes, and potential for integration into sensing platforms offer distinct advantages that could redefine how contaminants are detected and quantified, particularly under demanding conditions and in complex matrices.

Taking this into account, the preference between organic and inorganic fluorescent sensors is not absolute but context-dependent. The selection between these sensor classes is rarely a one-size-fits-all decision; rather, it is dictated by the specific analytical context, including the nature of the target analyte (e.g., metal ions, VOCs, biological species), the environmental matrix (air, water, soil), and the operational parameters, such as the detection limits, temporal resolution, stability under field conditions, and integration with existing analytical platforms. For example, while organic fluorescent probes may excel in detecting specific biomolecules, with high selectivity in controlled laboratory settings, inorganic sensors often demonstrate superior resilience and reproducibility when exposed to fluctuating environmental conditions or long-term deployment scenarios.

Such nuances underscore the need to evaluate sensor technologies based not only on their intrinsic properties but also on their practical applicability within diverse environmental scenarios.

Looking forward, the convergence of organic and inorganic materials into hybrid nanosystems may offer transformative opportunities. This approach, grounded in rational nanomaterial design and interdisciplinary synthesis strategies, has the potential to yield next-generation sensors with superior selectivity, durability, and scalability. Continued research in this direction will be instrumental in aligning sensor development with global needs for accurate and sustainable environmental diagnostics.

## Figures and Tables

**Table 1:** Most recent fluorescent sensors classified by organic materials, target, technique, type of sample and LOD.

Organic Material	Target	Technique	Type of Sample	LOD	[Ref.]
Small molecules					
*Pyrene-derivatives*	Ag^+^	Turn-off	HEPES-buffered DMSO/H_2_O	2.9 nM	[35]
	Hg^+^	Quenching	MeCN/H_2_O	4 ppb	
	Pb^2+^	Quenching	MeCN/H_2_O	2 ppb	
*Fluorescein*	Cu^2+^	Turn-on	Aqueous solution	6.32 nM	[40]
	Hg^2+^	Turn-on	Aqueous solution	0.86 nM	[41]
	ClO^−^	Turn-on–turn-off	Aqueous solution	56 pM	[42]
*Rhodamine*	Cu^2+^	Turn-on	MeCN/H2O	0.107 µM	[43]
*Cyanine dyes*	Hg^2+^	Color change	Acetonitrile	31 nM (naked eye detection: 2.9 ppm)	[51]
	Cu^2+^	Quenching	Acetonitrile	37 nM, (naked eye detection: 2.1 ppm)	
* BODIPY *	Dicofol	turn-off fluorescence	Water and tea	200 ppb	[53]
	Cd^2+^	color change		2.8 ppb	[54]
Nanoparticles					
*Carbon quantum dots*	Diazinon	Quenching	Cherry tomato juice	0.00821 µM	[63]
	Glyphosate	Quenching	Cherry tomato juice	0.00296 µM	[63]
	Fenitrothion	Quenching	Rice samples	0.00036 µM	[64]
	Malathion	Quenching	Water	0.00514 µM	[65]
	Chlorpyrifos	Quenching	Water	0.00427 µM	[65]
CQDs modified with Eu(III) complexes	Hg^2+^	Quenching	Milk	0.2 nM	[62]
N and Cl co-doped lignin CQDs	Polystyrene microplastics	Fluorescence emission	Water	0.4 mg/L	[66]
*Polymeric nanoparticles*					
Poly(DVB-co-TPE-PBE)	Picric acid	Quenching	Water solutions	5.43 µM	[73]
Poly(methyl methacrylate-co-glycidyl methacrylate) NPs	Fe^2+^	Color change and fluorescence emission enhancement		2.63 µM	[74]
	Fe^3+^			2.5 µM	
*Organic semiconducting nanoparticles (OSNs)*					
PCDA-Alen (based on PDA)	Pb^2+^	Color change (from blue to red) with increase in fluorescence output	Water from various natural bodies	16.3 nM (3.2 ppb)	[82]
TCDA-clay-N-1-hexadecyl imidazole	Toluene	Color change (from blue to red) with increase in fluorescence output	VOC atmosphere in sealed chamber (25 mL)	0.02%	[83]
	THF			0.08%	
	Benzene			0.04%	
Polymers					
*PPy*	Cu^2+^	Quenching	Water	1.2 μM	[90]
* PANI *	Malathion	Quenching	Potatoes, tomatoes	0.132 μM	[92]
*PIN*	Picric acid	Quenching	Organic media	30 × 10^3^ M^−1^ (*)	[96]
*PTh*	Berberine hydrochloride (alkaloid)	Decrease in fluorescence emission and color change (from pale yellow to red)	Water, urine samples	0.27 μM	[99]
* COPs *	Hydrazine	Fluorescence turn-on	Aqueous solutions	0.16 μM	[103]
*Conjugated polymers with N-heterocyclic moieties*	2,4,6-trinitrophenol (TNP) or picric acid solution and vapor	PL quenching	Aqueous solutions	147 nM (38 ppb)	[110]
* PVA *	Cu^2+^	Luminescence quenching	Aqueous environments	0.086 μM	[113]
* PS *	Hg^2+^	Increase in fluorescence emission	Real water samples	1.01 μM	[114]
*PMMA*	Chloroform	Luminescence quenching	Gas chamber	15.4 ppm	[117]
*PVAc*					
COFs					
COF-DHTA	Al^3+^	Turn-on	DMF suspension	0.93 μmol/L	[134]
COF-CB	Pb^2+^	Turn-on	DMF solution	1.48 µmol/L	[135]
	Hg^2+^	Quenching	Aqueous solution	17 nM	[180]
DHB-TFP COF	NP	Turn-off		0.40 μmol/L	[136]
	TNP (picric acid)	Turn-off		11.15 μmol/L	
TFPB-TTA COF	DNP	Quenching	Aqueous solution	18 nM	[181]
	TNP			16 nM	
MOFs					
MB@Cd-MOF	Carbaryl	Fluorescence enhancement	Tap and river water, fruit juices	6.7 ng·mL^–1^	[144]
TbMOF	Imidacloprid	Quenching	Water	1.3 × 10^−5^ M^−1^	[182]
	Thiamethoxam	Quenching		7.3 × 10^−6^ M^−1^	
{(Me2 NH2)[In(BDPO)]·DMF·2H 2O}n	2,6-Dichloro-4-nitroaniline (DCN)	Quenching	Water	0.14 μmol L^−1^ 3.85 ppm	[183]
Co-MOF	p-Nitrophenyl phosphate (PNPP)	Quenching	Food, fruits, and domestic water	352 nM	[184]
Cd(II)-MOF	Glyphosate	Turn-on fluorescence	Drinking water	0.025 μmol L^−1^	[185]
	Cr^3+^	Turn-on fluorescence		0.6 μM	
ZnMOF	Parathion	Quenching	Irrigation water	1.95 mg L^−1^	[186]
Zr-MOF	Monocrotophos (MCP)	“Turn-on” fluorescence	Tap water, lake water, wastewater	1.84 nM	[145]
Eu-MOF	Tetrahydrofuran (THF) vapor	Turn-on	THF-saturated air	17.33 Pa	[146]
Eu-MOF	Benzaldehyde solution		Benzyl alcohol	9.3 × 10^−6^ M	[187]
	Fe^3+^		DMF solution	5.8 × 10^−6^ M	
Dye@Eu-MOFs)	Acetaldehyde vapor	Quenching	Dye@Eu-MOF hydrogel plate	8.12 × 10^−4^ mg/L	[188]
ZIF-90 MOF	Formaldehyde	Turn-on	Liquid and gas phases	2.3 µM	[189]
Tb(BTC)-MOF	TNP vapor	Quenching	Saturated glass vial	<1 ppb	[190]
CuMOF	2,4,6-Trinitrophenol (TNP)	Quenching	River and tap water samples	0.08 μmol L^−1^	[191]
P1@BMOF	Cu^2+^	Quenching	Deionized water	0.22 μM	[192]
P2@BMOF				0.20 μM	
Eu@UiO-MOF-X	Cd^2+^	I_Eu3+/_I_BPYDC_ (663 nm/426 nm) reduction	Rice, grape juice, and liquor samples	5.67 × 10^−7^ M (114 ppb)	[193]
{[H-Phen]_2_[Mn_3_(FDA)_4_(H_2_O)_2_]·2H_2_O}_n_ (Mn-based MOF)	Ag^+^	Turn-on	Water	0.023 ppb	[194]
	Cd^2+^	Turn-on		0.05 ppb	
	Hg^2+^	Turn-off		0.10 ppb	
ZnTCPP-MOF	Pb^2+^	Quenching (from bright red to colorless under UV light)	Water	4.99 × 10^−8^ M	[195]
Biomolecule-based sensors					
*Protein-based fluorescent sensors*					
amGFP (sensor and bio-cleaner)	Cu^2+^	Quenching	Wastewater	1–5 μM (estimated)	[153]
*Enzyme-based fluorescent sensors*					
AuNC@ZIF-8	Acephate	Turn-on	Water, lettuce	0.67 μg/L	[155]
	Glyphosate			-	
	Malathion			-	
	Parathion			-	
	Pirimiphos-methyl, fenitrothion			-	
	(OPs)			-	
*Nucleic acid-based fluorescent sensors*					
G-CD@Apt	SARS-CoV-2 spike protein	Turn-on	Synthetic saliva, river water	0.067 ng/mL (equivalent to 0.335 pg per test)	[160]
Thrombin-binding aptamer–crystal violet (TBA-CV)	Pb^2+^	Fluorescence intensity decrease	Pond water	1.18 nM (0.32 ppb)	[196]
DNA nanosphere-enhanced substrate strand–DNAzyme (DS-Sub-Dz)	Pb^2+^	Fluorescence intensity increase	Drinking water, tap water, rainwater, and lake water	2.0 nM	[197]
DVc1 (DNAzyme)	* Vibrio cholerae *	Turn-on	Raw choking sea crab forceps, raw choking oysters, and cold jellyfish	7.2 × 10^3^ CFU/mL	[198]
*Antibody-based fluorescent sensors*					
Au-Ag bimetallic nanoclusters (NCs)	Dicofol	Fluorescence recovery	Green tea, black tea, and dark tea	0.185 ng/mL in liquid system	[162]
Gold nanoflowers (NFs)				0.170 ng/mL in paper-based analytical devices	
FPIA (composed of anti-IMI monoclonal antibody and fluorescein isothiocyanate ethylenediamine (EDF))	Imidacloprid (IMI)	Fluorescence polarization	Paddy water, corn, and cucumber	1.7 µg/L	[163]
*Fluorescent sensors based on living (micro)organisms or parts of them*					
*Whole-cell biosensors*					
*E. coli* S17-1 (donor)	1,3-Dinitrobenzene (1,3-DNB)		Liquid solution	0.1 μg/mL	[199]
*P. putida* (recipient)			Sand	0.5 mg/kg	
*Escherichia coli* DH5α	Hg(II)	Increase in red fluorescence	Cosmetics	0.03 μM	[200]
*Escherichia coli* DH5α	Pb (II)		Synthetic wastewater	2 nM	[201]
*Arxula adeninivorans*	17b-Estradiol	Fluorescence emission	Serum samples	1 ng/L	[202]
	Progesterone			6 ng/L	
	5a-Dihydrotestosterone			25 ng/L	
*Chlamydomonas reinardtii* *Organelle-based biosensors* Thylakoid membranes	Atrazine	Fluorescence yield increase Fluorescence decrease	Water Fish, milk, river water	7.3 × 10^−10^ M	[167]
	Diuron			2.3 × 10^−10^ M	
	Prometryn Netilmicin			3.5 × 10^−10^ M 5.99 nM [153]	

(*) expressed as Stern–Volmer constant (*K*_sv_).

## Data Availability

Not applicable.
